# Evaluation of an Air Cleaning Device Equipped with Filtration and UV: Comparison of Removal Efficiency on Particulate Matter and Viable Airborne Bacteria in the Inlet and Treated Air

**DOI:** 10.3390/ijerph192316135

**Published:** 2022-12-02

**Authors:** Peiyang Li, Jacek A. Koziel, Nubia Macedo, Jeffrey J. Zimmerman, Danielle Wrzesinski, Erin Sobotka, Mateo Balderas, William B. Walz, Reid Vincent Paris, Myeongseong Lee, Dongjie Liu, Bauyrzhan Yedilbayev, Brett C. Ramirez, William S. Jenks

**Affiliations:** 1 Department of Agricultural and Biosystems Engineering, Iowa State University, Ames, IA 50011, USA; 2 Livestock Nutrient Management Research Unit, USDA-ARS Conservation & Production Research Laboratory, Bushland, TX 79012, USA; 3 Department of Veterinary Diagnostic and Production Animal Medicine, Iowa State University, Ames, IA 50011, USA; 4 Department of Mechanical Engineering, Iowa State University, Ames, IA 50011, USA; 5 Department of Statistics, Iowa State University, Ames, IA 50011, USA; 6 Department of Animal Science, Texas A&M University, College Station, TX 77843, USA; 7 Department of Biomedical Sciences, Iowa State University, Ames, IA 50011, USA; 8 Department of Geography and Environmental Sciences, al-Farabi Kazakh National University, Almaty 050040, Kazakhstan; 9 Department of Chemistry, Iowa State University, Ames, IA 50011, USA

**Keywords:** indoor air quality, biosecurity, ultraviolet light, UVGI, UV254, UV disinfection, disease control, occupational health, air pollution control

## Abstract

Since the COVID-19 pandemic, improving indoor air quality (IAQ) has become vital for the public as COVID-19 and other infectious diseases can transmit via inhalable aerosols. Air cleaning devices with filtration and targeted pollutant treatment capabilities can help improve IAQ. However, only a few filtration/UV devices have been formally tested for their effectiveness, and little data is publicly available and UV doses comparable. In this research, we upgraded a particulate matter (PM) air filtration prototype by adding UV-C (germicidal) light. We developed realistic UV dose metrics for fast-moving air and selected performance scenarios to quantify the mitigation effect on viable airborne bacteria and PM. The targeted PM included total suspended particulate (TSP) and a coarse-to-fine range sized at PM_10_, PM_4_, PM_2.5_, and PM_1_. The PM and viable airborne bacteria concentrations were compared between the inlet and outlet of the prototype at 0.5 and 1.0 m^3^/s (low and high) air flow modes. The upgraded prototype inactivated nearly 100% of viable airborne bacteria and removed up to 97% of TSP, 91% of PM_10_, 87% of PM_4_, 87% of PM_2.5_, and 88% of PM_1_. The performance in the low flow rate mode was generally better than in the high flow rate mode. The combination of filtration and UV-C treatment provided ‘double-barrier’ assurance for air purification and lowered the risk of spreading infectious micro-organisms.

## 1. Introduction

Indoor air quality (IAQ) is crucial to human and animal health in residential and work-related settings. Poor IAQ can lead to the transmission of a variety of infectious airborne micro-organisms. Since the COVID-19 outbreak, the importance of IAQ has become increasingly critical, e.g., the SARS-CoV-2 virus and influenza A virus are transmissible via aerosols [1,2]. Therefore, properly cleaning indoor air can help mitigate disease transmission and improve health conditions. Two common methods of controlling indoor infectious aerosols are source control (e.g., physical distancing) and engineering control (such as filtration) [3]. This research focuses on the engineering control of PM and viable airborne bacteria.

The Centers for Diseases Control and Prevention (CDC) suggested higher air changes per hour (ACH) and enhanced filtration to reduce airborne disease transmission [4]. ACH is a metric associated with IAQ. In principle, a greater number of ACH makes for better IAQ because it replaces ‘dirty’ indoor air with ‘cleaner’ outdoor at a faster rate. In some cases, it is not practical to increase room ventilation rates to enhance ACH due to constraints in existing heating, ventilation, and air conditioning (HVAC) systems. Under such constraints, air cleaning devices (portable or fixed) equipped with filters and other air cleaning technologies can improve IAQ by continuously recirculating and cleaning the air to achieve an “air changes per hour equivalent” (ACHe).

Air-cleaning devices commonly use filters to filter out particulate matter (PM) and viable airborne bacteria (bacteria, fungi, and viruses). The clean air delivery rate (CADR) is generally referred to as the volumetric flow rate of ‘cleaned’ (treated or filtered) air that moves through air treatment devices (such as filters) to determine the contaminant (such as smoke and dust) removal effectiveness [5]. The CADR value is useful in establishing whether a particular device can achieve the target ACHe in a designated space [6,7]. Many centralized mechanical ventilation systems use lower-grade filters (MERV-8 or less) for air cleaning, which may be insufficient to remove viable airborne bacteria and finer PM effectively [6].

According to the American Society of Heating, Refrigerating and Air-Conditioning Engineers (ASHRAE) Standard 52.2 [8], MERV-8 filters capture >20% of particles 1.0–3.0 µm in diameter and >70% of particles 3.0–10.0 µm but are not effective for particles 0.30–1.0 µm. Higher grade filters, e.g., MERV-13, remove ≤50% of particles 0.3–1.0 µm and ≥85% of particles 1.0–3.0 µm in diameter [8]. To aim for a higher pollutant removal rate, higher MERV rating filters may be installed in combination with prefilters (such as MERV-8) to capture finer PM, such as airborne bacteria and viruses, and other air treatment technologies can be added as secondary treatments.

For contextual comparison with non-viable PM, a common bacterium, *Escherichia coli* (*E. coli)*, is a cylindrical rod with 1.1–1.5 µm in diameter and 2.0–6.0 µm in length [9], and larger bacteria may be a few µm in diameter. Typical human viruses, e.g., influenza A virus, are about 0.1 µm in diameter [10]. Note that viable airborne bacteria most commonly exist as clumps and/or attached to larger particles. Qian et al. (2012) [11] reported that bacterial concentrations in an occupied classroom were predominantly found on airborne PM that is 3–5 µm in diameter.

Ultraviolet-C light (UV-C, commonly in 254 nm) has been used for decontamination and disinfection in various applications [12], including indoor air cleaning. The germicidal effectiveness of UV-C on airborne human and animal pathogens, including the recent coronavirus, has been proven [13,14,15]. Filtered far UV-C (207–222 nm) has also been shown to be germicidal and claimed to be less hazardous [16]. Longer UV wavelengths, e.g., UV-A, can be germicidal, especially in photocatalytic applications [17]. However, UV-A is not strongly absorbed by genetic material (DNA and RNA) and requires more than a magnitude of order higher dose than UV-C, resulting in higher capital and operational costs.

A variety of indoor air cleaning devices are commercially available, with some incorporating UV technology. However, many have not been independently tested and quantified for their effectiveness. Some may have undergone testing, but the results are not publicly available. We completed preliminary, internet-based market research on more than 50 portable air cleaners with filtration and/or UV technologies in 2021. We found that few of the air-cleaning products provided sufficient information that can prove their air-cleaning effectiveness, such as CADR and estimated UV dose (if UV lamps are used). Selected few product’s fact-sheets mentioned the theoretical filtration efficiency (%) on PM but were not specific on testing results. On the other hand, it should also be recognized, however, that testing under laboratory conditions (PM and pathogen level) may not predict the performance of the air-cleaning device in practical applications. Küpper et al. (2019) [18] acknowledged that the tested air cleaner had lower efficacy in realistic situations than in standardized testing situations. Testing in realistic scenarios that simulate the high loading of PM and pathogens in potential indoor spaces can be beneficial in assessing the performance of air-cleaning devices under harsh conditions. Additionally, testing in human or animal housing locations can confirm the effectiveness of adapting to variable air quality.

During the early peak of COVID-19, we collaborated with an Iowa-based manufacturing company to prototype and test a device for air treatment. We upgraded a PM-only air filtration prototype designed for large factory floors, warehouses, and public spaces and added UV-C (germicidal) light. Herein, we only focus on upgrading and evaluating the prototype performance based on the two critical metrics for which it was designed to mitigate: PM and airborne pathogens. Therefore, the objectives of this research were to (1) evaluate, design, and upgrade an existing air filtration device with the addition of UV-C lamps; (2) improve the measurement and estimate of UV-C doses needed for SARS-CoV-2 inactivation based on literature data; (3) testing the effectiveness of the upgraded device in inactivating viable airborne bacteria and PM in realistic conditions.

## 2. Materials and Methods

### 2.1. Upgrade of an Air-Cleaning Prototype

The FastAir prototype (2.54 × 1.52 × 1.02 m, 100 × 60 × 40 in.) was initially designed and manufactured by Kryton Engineered Metals Inc. (Cedar Falls, IA, USA) for filtering out PM generated during manufacturing and welding in indoor spaces. The original prototype without UV is shown in Figure S1 in the Supplementary Materials. Inside the prototype, an air blower was connected by a belt to a motor with two flow rate modes, termed low (~0.5 m^3^/s) and high (~1.0 m^3^/s). There were two layers of filters stacked inside the chassis, MERV-8 filters (51 × 61 × 10 cm, 20 × 24 × 4 in.) (Air Handler manufactured for W.W. Grainger, Inc., Lake Forest, IL, USA) on the outside and MERV-13 filters (fiberglass pocket filters, 51 × 61 × 38 cm, 20 × 24 × 15 in.) (Air Handler manufactured for W.W. Grainger, Inc., Lake Forest, IL, USA) on the inside (Figure S2).

**Figure 1:**
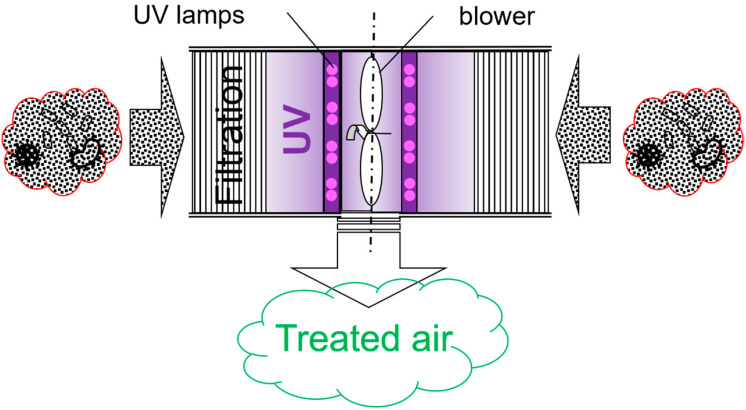
FastAir prototype for air treatment (top view). Untreated air enters on both sides and is filtered and irradiated by UV-C lamps. The air blower facilitates untreated air suction and ejection on treated air.

We upgraded this prototype by incorporating UV-C lamps to enhance the air-cleaning effect, especially for viable airborne pathogens. We also rearranged the filters to create more space for placing UV treatment between the filters and the blower. A simplified conceptual schematic illustrating the air treatment of the upgraded prototype is shown in Figure 1.

**Figure 2:**
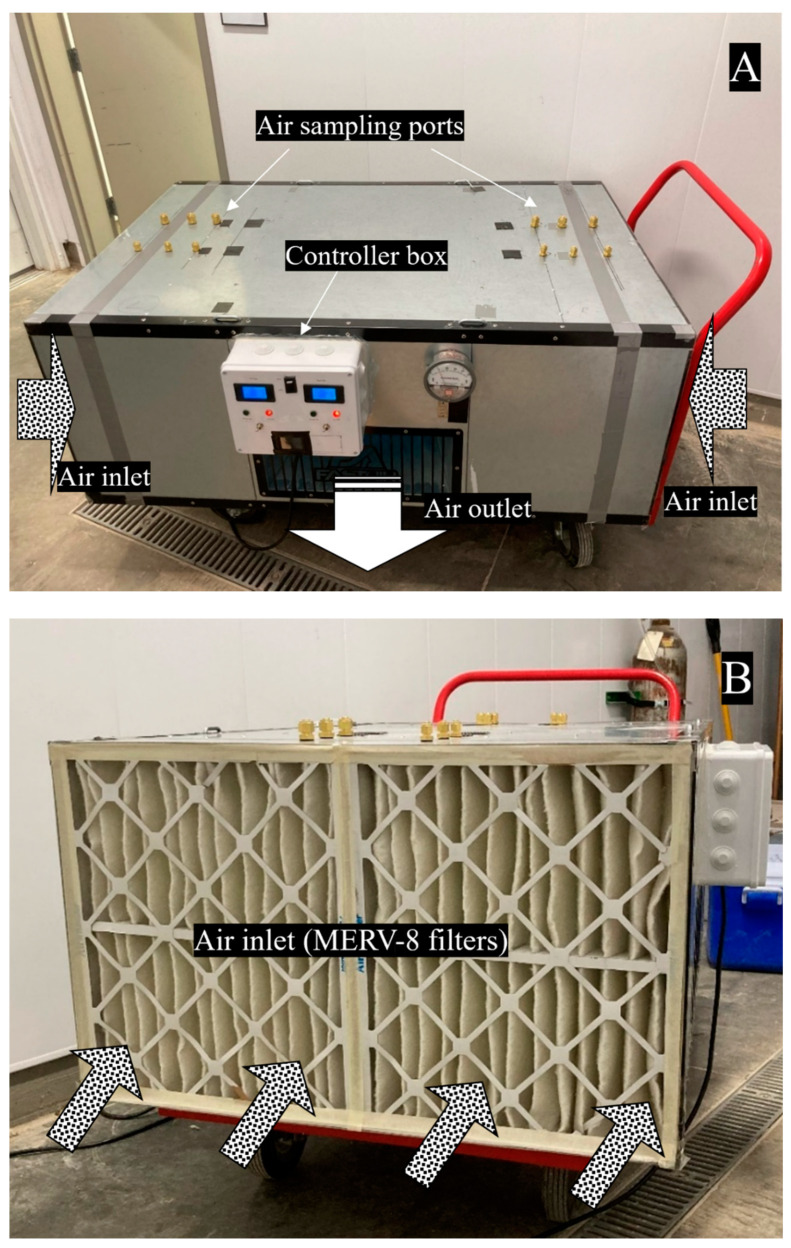

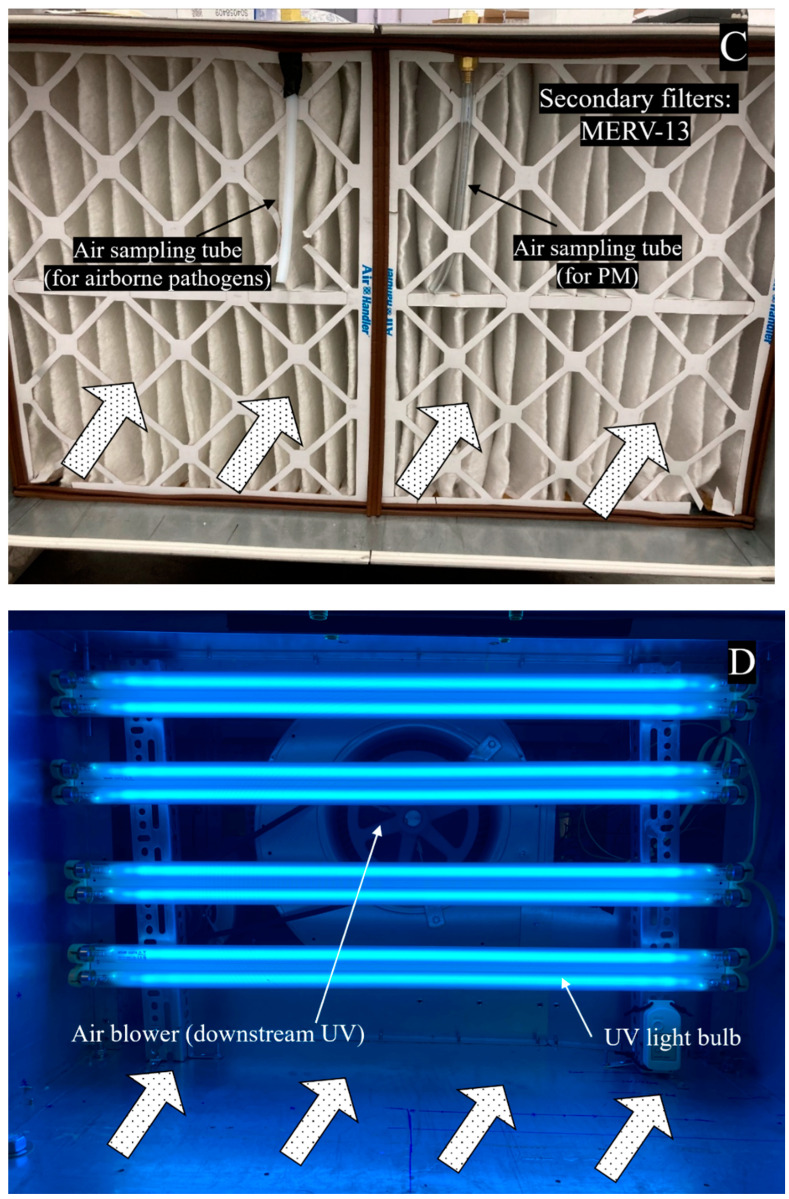
(**A**): Front view of the upgraded FastAir prototype with UV-C lamps enclosed and a controller box attached above the air outlet. (**B**): Side view of the upgraded FastAir prototype. MERV-8 filters can be seen at the air inlet. (**C**): After MERV-8 filters were removed, MERV-13 filters can be seen as the second filtration layer. Aluminum mesh filters follow MERV-13 filters to protect MERV filters from degradation caused by long-term UV irradiation. (**D**): Eight UV-C light bulbs were installed on each side of the FastAir prototype. Each light fixture supported twin UV-C light bulbs. The air blower can be seen downstream from the UV-C lamp. The gradient of the air flow arrows signifies progressively cleaner air (from (**B**–**D**)).

The upgraded prototype is shown in Figure 2A–D. The interior of the prototype was modified to accommodate the addition of UV-C lamps. The overview of the original and upgraded configurations are shown in Figures S3 and S4, respectively. MERV-8 filters on the outmost layer were unchanged. The modifications included replacing the 0.38 m (15 in.) deep fiberglass MERV-13 pocket filters with pleated 0.10 m (4 in.) deep MERV-13 filters, adding aluminum (Al) mesh filters, selecting and installing UV-C light bulbs and fixtures that provided a UV dose consistent with recommendations for SARS-CoV-2 inactivation (as described in Results Section 3.5). The reason for choosing narrower MERV-13 filters was to add space to install UV light and ensure sufficient treatment time for inlet air under UV irradiation. Because long-time exposure to UV can degrade filter fibers, an aluminum mesh filter (51 × 61 × 5 cm, or 20 × 24 × 2 in.) (All-Filters, Inc., Reno, NV, USA) was selected and placed between the UV light and filters to allow sufficient air flow while blocking the majority of UV from irradiating the MERV filters (Figure S5). A controller box that controls the electric power of the blower and UV light was attached to the prototype chamber (Figure S6).

### 2.2. UV Irradiance and UV Dose Estimation

After comparing several major UV lamp products, we selected low-pressure mercury bulbs for the FastAir prototype due to their wide availability and lower installation and replacement costs. Eight (8) UV-C (254 nm) low-pressure mercury bulbs (G30T8, Ushio America Inc., Cypress, CA, USA) in four (4) fixtures (Lithonia Lighting, Atlanta, GA, USA) were mounted on each side of the FastAir prototype, with each fixture containing two bulbs. In total, 16 UV bulbs and eight fixtures were installed on both sides of the FastAir prototype. Each bulb was 90 cm (36 in.) long and 2.5 cm (1 in.) in diameter and had a nominal power consumption of 30 W and a nominal UV output of 13.9 W. The average life of the UV bulb selected is about 8000 h in operation, according to manufacturer information [19]. The length of the bulb was close to the width (~1 m) of the FastAir prototype, as the goal was to maximize the range of direct irradiation on treated air. The total UV power consumption for the 16 bulbs, measured using a power meter, was about 350 W during regular operation.

#### 2.2.1. UV Light Measurement

UV-C irradiance (light intensity) was measured using an ILT 1700 radiometer (International Light Technologies, Peabody, MA, USA) with an NS240 light detector and SED240 filter, with a center wavelength of 254 nm and a tolerance range of 254~257 nm. The average UV irradiance measurements on virtual vertical (cross-sectional) planes parallel to UV lamps were performed on a 7 × 5-point gridded plane (each grid was 0.14 × 0.12 m). Figure S7 shows the UV detector mounted on steel support that can be moved based on the distance and location of the grids. Each measurement took place at the center of each grid.

**Figure 3:**
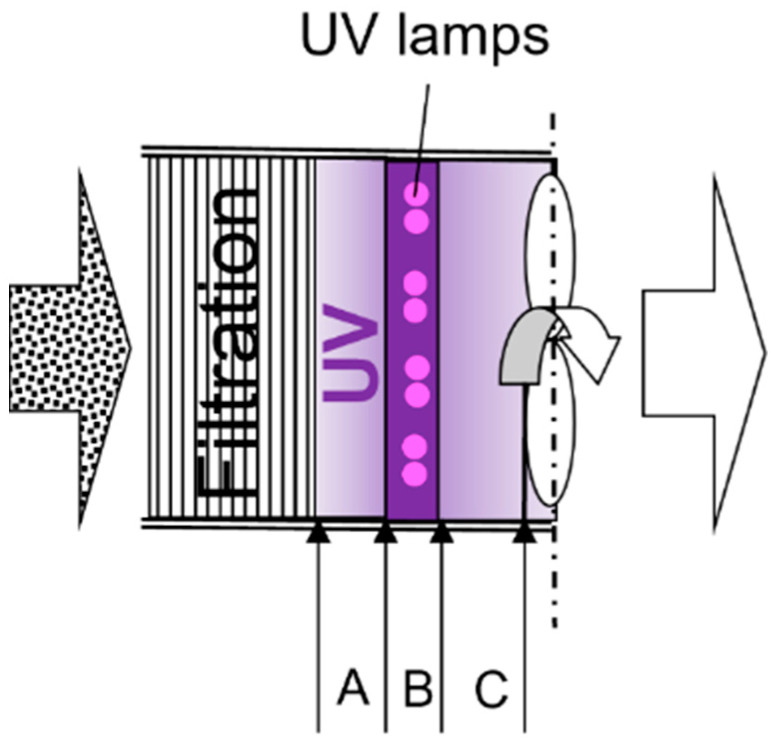
The schematic of UV irradiation inside the FastAir prototype. The filtered air entered three UV treatment zones, A, B, and C (upstream from lamps, in the near vicinity of lamps, and downstream of lamps).

Figure 3 shows the UV irradiation inside the FastAir prototype. After passing the “filtration” section, fast-moving air entered Zone A, B, and C, respectively. Zone A referred to the space upstream of UV lamps; Zone B referred to the space of UV lamps (in the near vicinity of bulbs and fixtures); Zone C referred to the space downstream of UV lamps and before the inlet to the blower. The averaged total UV treatment time was estimated to be 0.36 s and 0.74 s for high and low flow rate modes, respectively, based on the air flow rates and total volumes of Zones A–C.

#### 2.2.2. UV Irradiance and Dose Estimation

Most literature reports laboratory-scale studies where the UV doses needed for the inactivation of pathogens are on a 2-dimensional (2-D) scale in terms of UV energy per unit area. On the other hand, it appears to be that the data on a 3-D scale (UV energy per unit volume) is not available. Yet, in this UV treatment application, it is the volume of air that is being decontaminated, not the surface area. However, it is still necessary and appropriate to use, design, and compare based on the UV dose estimations on a 2-D scale for pathogens of interest in peer-reviewed literature. In addition, we estimated the UV dose in 3-D to account for the nature of the study in a fast-moving volumetric flow rate. In total, we estimated the UV dose in three ways (each including irradiated surface area- and volume-based estimations). Regardless of the approach, the basic theory is briefly introduced below.

Most currently available literature on UV dose for microbial inactivation for static (e.g., surface, liquid) or dynamic (e.g., air) scenarios recorded UV dose on a 2-D scale (i.e., energy per unit area, in mJ/cm^2^ or J/cm^2^) [20,21] using the Bolton and Linden equation (2003) [22] as shown as Equation (1) below. Bolton and Linden proposed the term UV dose to describe the total UV radiant energy absorbed per unit area:

(1) D=I×t

where D is the UV dose (mJ/cm^2^), I is the irradiance or light intensity (mW/cm^2^), and t is the UV treatment time (s).

To be consistent and comparable with similar research on UV inactivation, we conducted UV dose calculation on a 2-D scale. In addition, we realized the difference between static UV inactivation (such as on Petri dishes) and volume-based UV inactivation (such as on aerosols). Therefore, we added volumetric UV dose estimations. We estimated the UV dose in three ways (from virtual plane-based to volume-based approaches). Because Equation (1) was introduced for 2-D UV inactivation, some adjustments were made to fit each method.

##### Method 1: Estimation by UV Energy Output

*2-D area-based estimation*

This method estimated the UV dose based on the manufacturer’s specification of UV lamp power output (wattage) on an imaginary, vertical plane perpendicular to air flow. We introduced Equation (2) to estimate the UV dose imposed on fast-moving air inside the FastAir prototype.

(2) $$D=\frac{P_{UV}}{A}\times t$$

where D is the UV dose (J/m^2^), P_UV_ is the total UV power output (W) which equals the nominal output of a single UV bulb multiplied by the number of bulbs, A is the cross-section area (m^2^) of irradiation perpendicular to the air flow, and t is the average total treatment time (s).

Each UV bulb nominally consumes 30 W, of which 13.9 W is UV output, according to the manufacturer. This method assumes an ideal case, i.e., no light loss, ideal UV irradiation, and uniform air flow distribution. Therefore, the UV dose delivered to treated air was 12.8 mJ/cm^2^ and 6.3 mJ/cm^2^ (converted to mJ/cm^2^ for comparison purposes), respectively, for low and high air flow rate modes.

*3-D volume-based estimation*

Alternatively, Equation (2) can be written into Equation (3) below to account for the volumetric air flow rate to yield a 3-D UV dose (energy per volume). Considering the UV irradiance was for gas phase (i.e., air) irradiation rather than liquid or solid phase (i.e., surface) irradiation, an air volumetric flow-based UV dose would also be applicable in estimating the UV dose for air disinfection. This method utilized the UV energy output mentioned above but incorporated the volumetric air flow rate (in 3-D) rather than the air velocity (in 2-D) to calculate the UV dose on a volumetric basis, as described in Equation (3).

(3) $$D_{vol}=\frac{P_{uv}}{Q}$$

where D_vol_ is the volumed-based UV dose (J/m^3^), P_UV_ is the total UV power output (W) which equals the nominal output of a single UV bulb multiplied by the number of bulbs, Q is the volumetric air flow rate (m^3^/s) corrected to dry, standard condition (1 atm at 20 °C).

This method also assumed no light loss, ideal UV irradiation, and uniform air flow distribution. Therefore, the calculated UV dose was 0.92 mJ/cm^3^ and 0.45 mJ/cm^3^, respectively, for low and high air flow rate modes.

##### Method 2: Estimation by Measured Irradiance of a Single Plane

*2-D area-based estimation*

This method calculated the UV dose directly using Equation (1). The average UV irradiance was measured on a vertical, virtual plane, as detailed in Section 2.2.1. The average distance between the edge of the UV bulbs and the UV detector during measurement was 13 cm (5 in), which was assumed to be the maximum range the UV irradiation could reach inside the FastAir prototype, as indicated in Figure 3. The average UV irradiance was 4.2 mW/cm^2^ for the measured plane. Therefore, the UV dose delivered to treated air was 3.1 mJ/cm^2^ and 1.5 mJ/cm^2^, respectively, for low and high air flow rate modes.

*3-D volume-based estimation*

Using the same measured irradiance above, the total UV energy output was estimated, assuming the UV irradiation was equally distributed on two planes (upstream and downstream). The estimated UV dose based on Equation (3) was 0.22 mJ/cm^3^ and 0.11 mJ/cm^3^, respectively, for low and high air flow rate modes.

##### Method 3: Estimation by Integration of Measured Irradiance across Multiple Planes

*2-D area-based estimation*

In a method description regarding the measurement of irradiance output of monochromatic (254 nm) low-pressure UV lamps, Lawal et al. (2017) [23] and ISO (2020) [24] pointed out that to make sure that the UV detector can capture the entire length of the UV lamp, the distance between UV light to the detector should be at least twice as the length of the lamp. However, in this research, we are not interested in measuring the total UV irradiance output that far away (2 × 0.3 = 0.6 m), as the UV irradiation space inside the FastAir prototype is only in the magnitude of centimeters. Therefore, a different procedure was developed to offset the limitation of near-distance UV measurement (Figure S8). Building upon Equation (1) and considering the limitation of the UV detector in near distance to lamps measurement, we proposed Equation (4) that provides a more realistic estimate for the cumulative UV dose.

(4) $$D_{cum}=\sum_{1}^{n}I_{n}t_{n}$$

where D_cum_ is the cumulative UV dose in the irradiance space; n is the number of planes of measurement; I_n_ is the average irradiance at the plane parallel to the plane of lamps, and t_n_ is the estimated time for air that passes between two adjacent planes.

Equation (4) estimates the cumulative UV dose by integrating the measured UV doses of multiple virtual planes with the distance between each = 2.5 cm (1 in.). The proposed approach is more representative of the overall UV irradiance, treatment time, and, therefore, the cumulative UV dose, as compared with measured UV irradiance on a singular cross-sectional plane and a selected distance from lamps, as described in Method 2.

The graphical representation of the application of Equation (4) is presented in Figure 3. The cumulative UV dose was estimated as the treated air passed through three distinct zones. Zone A was between the Al mesh and the front of the UV lamps, i.e., the average distance for the air to pass through it was ~7.5 cm (3 in.). The UV irradiance in Zone A was measured every 2.5 cm (1 in.), and the total UV dose in Zone A was the integration of these three cross-sectional layers with the estimated average time of air passing through. Zone B was in the immediate vicinity of UV lamps. It was ~6.3 cm (2.5 in.) wide, and the UV irradiance measured at 2.5 cm (1 in.) away from the lamp was used to represent this zone for UV dose estimation. Zone C was the gap between the back (downstream) of the UV lamp and the front edge of the blower, i.e., the average distance for the air to pass through it was ~13.8 cm (5.5 in.). Because it was not feasible to measure UV irradiance in Zone C, and the irradiance was lower due to the shadow of the lamp fixtures and its support, the zone was conservatively assumed to have the irradiance of the cross-sectional plane at the front edge of the blower (i.e., at 12.5 cm or 5 in. away from lamps). These estimates were likely underestimated due to limitations discussed earlier (Figure 3). Therefore, the UV dose delivered to treated air was at least 2.9 mJ/cm^2^ and 1.4 mJ/cm^2^, respectively, for low and high air flow rate modes. These values are within 10% difference with Method 2, which was much simpler to operate.

*3-D volume-based estimation*

Using the average UV irradiance (4.06 mJ/cm^2^) measured from several planes mentioned above, the total UV energy output was estimated, assuming the UV irradiation was equally distributed on two planes. The estimated UV dose based on Equation (3) in Method 2 is 0.22 mJ/cm^3^ and 0.11 mJ/cm^3^, respectively, for low and high air flow rate modes, which are approximately the same as the results of Method 2.

#### 2.2.3. Air Flow Rate Measurement and Conversion

A fan assessment numeration system (FANS) [25], a portable fan testing device, was used for air flow measurements. The FANS unit included a horizontal array of four propeller anemometers inside an aluminum frame to measure the real-time air flow traverse through the unit. Additionally, a 3 m (10 ft) rigid foam air duct that aimed to stabilize the turbulent flow was constructed to connect the outlet (smaller opening) of the FastAir prototype to the opening of the FANS unit (Figures S9 and S10). The volumetric air flow rate was measured before and after the UV upgrade was made. The measurements covered different conditions, i.e., loads of filters/light (all filters, no filters, MERV-13 only, etc.). The results of the volumetric air flow rate were converted to a dry-standard air flow rate at standard conditions (293.15 K or 20 °C) based on Equations (5) and (6), according to Hoff et al. (2009) [26].

(5) $$Q=\left(1-W\right)\frac{Q_{a}PT^{\prime }}{P^{\prime }T}$$

where Q is the dry standard air flow rate (m^3^/s); W is the absolute humidity ratio (kg_w_/kg_a_, i.e., water vapor to dry air mass ratio), which can be calculated in Equation (6) [26]; Q_a_ is the actual moist air flow rate (m^3^/s) measured by FANS unit; T′ is the standard temperature defined as 293.15 K; T is the actual dry-bulb temperature (K) during air flow measurement; P is the actual atmospheric pressure (Pa) during air flow measurement; P’ is the standard pressure, defined as 1 atm (101,325 Pa).

(6) $$W=0.62198\frac{P_{w}}{P_{a}-P_{w}}$$

where P_w_ is the partial pressure of water vapor during air flow measurement (Pa) using the methods described in Hoff et al. (2009) [26].

### 2.3. Experimental Design

#### 2.3.1. Testing Sites

The FastAir prototype was first tested in a laboratory room for air cleaning effects. However, due to the modern air cleaning equipment available in the building, the air in the laboratory was very clean (i.e., TSP concentrations were less than 10 µg/m^3^, and viable airborne bacteria were hardly recovered with SKC BioSamplers^®^), so the treatment effect could not be substantiated. A summary of the preliminary testing results is included in Tables S2 and S3 in the Supplementary Material. Considering this, we chose one of the poultry rooms at the Robert T. Hamilton Poultry Teaching & Research Farm as the testing site because the indoor air environment is dusty and contains a sufficient level of viable airborne bacteria; therefore, the treatment effect could be easily observed and quantified. The room selected for testing was 11.9 × 4.9 × 4.3 m (L × W × H), equipped with mechanical ventilation and a direct gas-fired circulating heater, and housed about 150 laying hens in conventional cages (Figure S11). During the experiment, the poultry room temperature ranged from 20.0 °C to 23.3 °C, and relative humidity (RH) ranged from 20% to 45%.

#### 2.3.2. Air Cleaning Configuration Options

The experiment aimed to evaluate the effectiveness of six different configuration options under both low and high air flow modes (Table 1). The “filtration + UV” option deployed both configurations simultaneously. However, we also evaluated the performance of “filtration only” and “UV only” options to estimate the level of air treatment redundancy and, therefore, the risk of prototype failure. Note that both types of filters were not replaced between experiments due to the lifespan of the filters (weeks or months of continuous operation) far exceeded the time used in the experiments (hours). However, the PM load on filters was periodically checked via visual inspection, and the pressure drop across the filters was monitored continuously.

Six configuration options were commissioned to test the FastAir prototype under high and low flow rate modes. They are denoted with the following acronyms (in parenthesis) for convenience: high mode, filtration + UV (H-F + UV); high mode, filtration only (H-F); high mode, UV only (H-UV); low mode, filtration + UV (L-F + UV); low mode, filtration only (L-F); low mode, UV only (L-UV).

**Figure 4:**
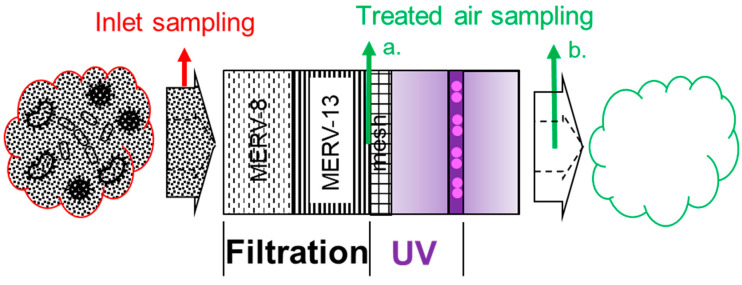
Details of FastAir prototype for air treatment (one symmetrical side shown) following the air flow direction. Treated air can be sampled after each treatment phase (e.g., filtration and UV) for PM and viable airborne bacteria. The red arrow indicates the air sampling location for the inlet (room air), and the green arrows indicate two air sampling locations for different configuration options: (a.) after “filtration only”, (b.) after “UV” or after “filtration + UV”.

**Figure 5:**
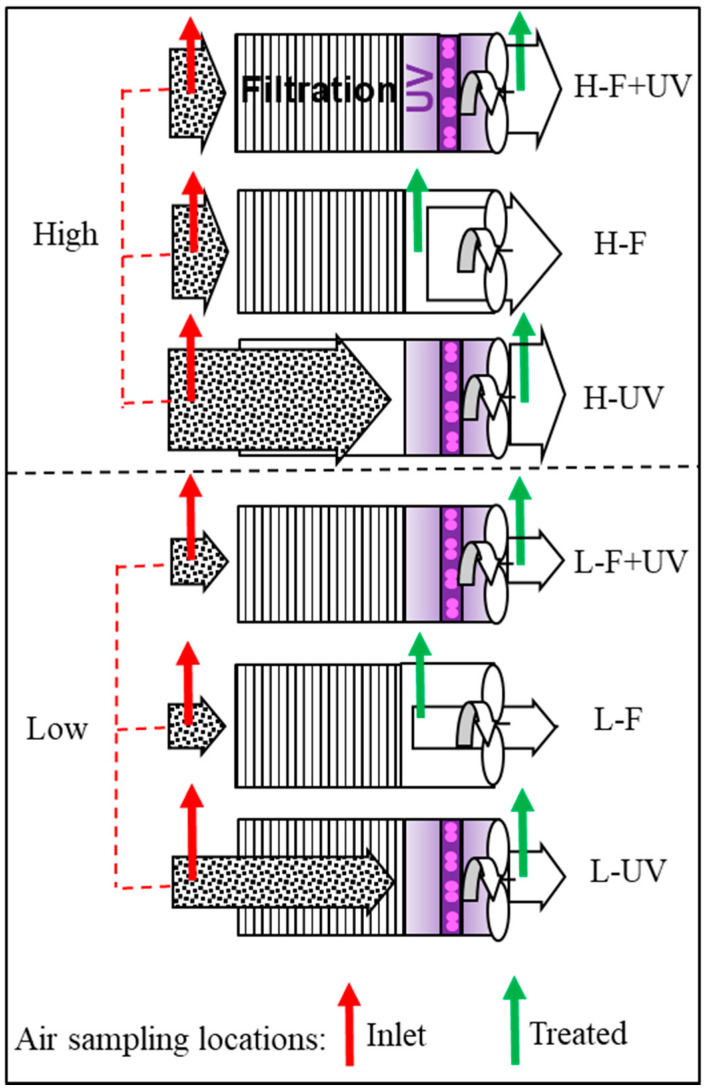
Six air cleaning configuration options were used for testing of FastAir prototype in high (top three panels) and low (bottom three panels) air flow rate modes, with both modes having filtration + UV, filtration only, and UV only options. Red and green arrows signify the sampling locations of the inlet and exhaust (treated) air, respectively, for PM and viable airborne bacteria. Each of the six configuration options was tested three (*n* = 3) times in experimental trials.

The location of air sampling ports of each configuration option is illustrated in Figure 4, and a complete list of schematics of all six configuration options is shown in Figure 5 and Figures S12 and S17.

In each option, inlet air was sampled near the inlet of the FastAir prototype, while treated air was sampled at the locations right after (downstream from) each specific configuration option (filtration and/or UV). Two parameters were measured and calculated to evaluate the performance of the prototype: PM (including PM_1_, PM_2.5_, PM_4_, PM_10_, and total suspended solids, TSP; unit: µg/m^3^) and airborne bacteria concentrations (colony-forming units per m^3^ of air, or CFU/m^3^). Each of the six configurations was tested three times (*n* = 3). Prototype performance was evaluated by the percent mitigation of PM and airborne bacteria between the inlet and treated air.

Twelve sampling ports (six on each side of the device) were made using tubing fittings (bulkhead union, Swagelok Company, Solon, OH, USA). The tube fittings for PM sampling fit for 9.5 mm (3/8 in) tube OD, and those for bacteria sampling fit for 13 mm (½ in.) tube OD. Tube inserts were installed for the PM tubing fittings to connect with Tygon tubing (7.9 mm, 5/16 in ID). For airborne bacteria sampling, stiff PTFE tubing (9.5 mm or 3/8 in ID, 12.7 mm (½ in) OD) was connected with Swagelok fitting. The sampling ports, tubes, and locations can be seen in Figures S16–S19. The sampling ports were located symmetrically between the two sides of the FastAir prototype. The sampling ports and tubes were located in pairs: (α) after the MERV-8 filter, (β) after the MERV-13 filters, and (γ) after aluminum mesh filters. Location (γ) was not used in the experiment. Location (α) was used during the experiment in the preparation phase, but the results were not included in the Results section as we focused on the collective effect of MERV-8 and MERV-13 filters. Based on the experimental design shown in Figure 5, location (β) was responsible for configurations (L-F and H-F). For the rest of the configurations, the air was sampled from the exhaust of the FastAir prototype directly. During each experiment, sampling ports that were not used were sealed with caps.

### 2.4. Air Quality Measurements

#### 2.4.1. Sampling and Recovery of Viable Airborne Bacteria

**Figure 6:**
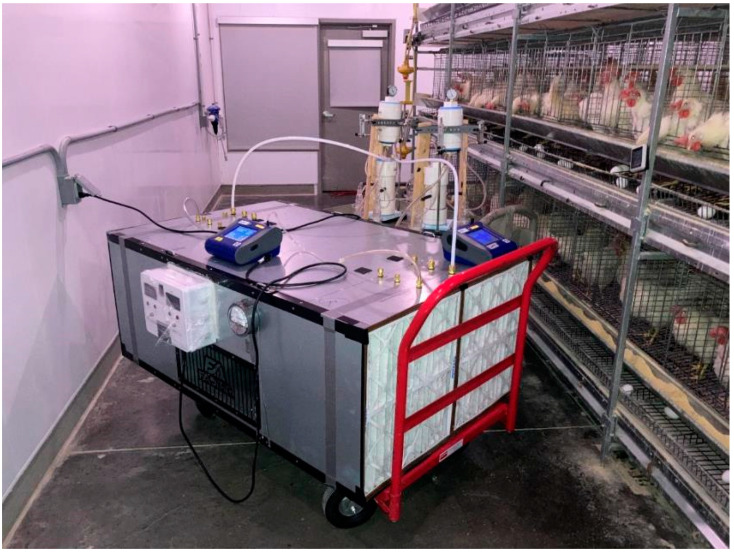
The experimental setup for collecting viable airborne bacteria and PM at a teaching room that housed ~150 laying hens at ISU Poultry Teaching and Research Facility. The red cart was used to deploy the FastAir prototype into the testing room. The brown cart (behind) held all sampling equipment (vacuum pumps, manifolds, and BioSamplers^®^).

The experimental setup for collecting viable airborne bacteria and PM is shown in Figure 6. Viable airborne bacteria were collected using SKC BioSamplers^®^ (20 mL) (SKC Inc, Eighty Four, PA, USA) [27]. Three BioSamplers^®^ (*n* = 3) were used for inlet and treated air, respectively, for simultaneous sampling. The duration of each trial was 1 h, and all BioSamplers^®^ were placed in an ice bath during the process (Figure S22). Each treatment option was run for at least three trials (*n* ≥ 3). Two sampling stations were constructed using common polyvinyl chloride (PVC) pipes (10 cm; 4 in for ID) and brass fittings (for 9.5 mm (3/8 in) hose ID; sourced from W. W. Grainger, Inc., Lake Forest, IL, USA) to facilitate the air impingement process (Figure S23). Briefly, each sampling station consisted of two parts: the vacuum section (top, ~35 cm tall) and the sampling section (bottom, ~30 cm tall). The top section included a triple-connector manifold that connects three rotameters (Catalog No. RMA-21-SSV, Dwyer Instruments Inc., Michigan City, IN, USA) that were positioned isometrically (120 deg apart from each other) to measure and control equal air flow distribution among three branches, a pressure gauge at the top to monitor the vacuum level, and a port connecting to the vacuum pump that provided suction to direct the air flow. The sampling section also had three isometric brass fittings with Tygon tubing attached that each could connect to the inlet of an SKC BioSampler^®^. Brass fittings were connected by autoclavable Tygon tubing (9.5 mm (3/8-in ID, 12.7-mm or ½-in OD). Two larger ports were installed on top of the sampling section as each port could connect with a polytetrafluoroethylene (PTFE) tube (9.5 mm (3/8 in) ID & 12.7 mm; (½ in) OD) for air sampling from the FastAir prototype. BioSamplers^®^, air sampling tubing, and connecting tubing were disinfected between experimental trials. The gauge vacuum on the sampling station connected to the BioSamplers^®^ was maintained at least at 0.5 atm (15 in Hg) to allow for proper air flow through the three tangential nozzles (performing as critical, sonic orifices) inside each BioSampler^®^.

Following manufacturer recommendations, the nominal readings on the rotameter scale were converted for the standard condition (defined as 20 °C and 1 atm). Equation (7) was used to convert the nominal flow rate reading operated in nonstandard conditions to the standard condition.

(7) $$Q_{s}=Q_{a}\;\sqrt{\frac{P_{a}\times T_{s}}{P_{s}\times T_{a}}}$$

where Q_s_ is the standard flow rate corrected for standard pressure temperature, Q_a_ is the actual or observed flow rate reading on the rotameter during operation, P_a_ is actual pressure (1 atm (14.7 psia) + gauge pressure) measured at the outlet of the rotameter, T_s_ is the standard temperature (293.15 K), P_s_ is the standard pressure (1 atm (14.7 psia), which is 0 psig), T_a_ is the actual temperature (unit: K).

Prior to the experiment, each BioSampler^®^ was filled with 20 mL of PBS (phosphate-buffered saline, 1×, pH = 7.4). Vacuum grease was used to seal the connecting area among the three parts of the sampler. The impingement process was driven by two oil-free rotary vane vacuum pumps (Becker VT 4.16 vacuum pump, Becker Pumps Corp., Cuyahoga Falls, OH, USA) (Figure S24). After sampling, the BioSampler^®^ liquid was collected, labeled, and sent to a bacteriology laboratory for culturing. A total plate count was performed to quantitate the colony-forming units (CFUs) per ml. The procedure was adopted with minor modifications from Laird et al. (2004) [28]. The sampling liquids from BioSampler^®^ were first centrifuged at 400× *g* for 10 min at room temperature to prepare the liquid needed for the colony counts. Most liquids were decanted, and approximately 1 mL was left and then homogenized by vortexing. Ten-fold serial dilutions were conducted by pipetting 0.10 mL of the sample liquid into 0.90 mL of PBS. Next, 0.1 mL of each dilution and the original, undiluted liquid were inoculated onto 5% sheep blood in tryptic soy agar (Hardy Diagnostics, Chicago, IL, USA) plates and spread on the agar with sterile loops to immobilize the pathogen cells on the agar surface allowing growth of distinct, non-overlapping colonies. All the agar plates were incubated aerobically at 37 °C for 48 h, and the bacterial colonies were counted at the specific dilution with visible 10–200 colonies (Figure S25). The raw data were expressed as CFU per milliliter of sampling fluid (CFU/mL).

The concentration of viable airborne bacteria in sampled air can be calculated:

(8) $$C=\frac{C_{s}\times V_{s}}{V_{a}}$$

where C is the concentration of viable airborne bacteria in the sampled air (CFU/m^3^), C_s_ is the concentration of pathogens in impinger liquid, vs. is the volume of sampling fluid (20 mL), V_a_ is the volume of air passing through the impinger in 1 h.

The percentage of mitigation for viable airborne bacteria or PM can be calculated by Equation (9):

(9) $$\%R=\frac{C_{c}-T_{c}}{C_{c}}$$

where %R is the percentage of mitigation, C_c_ is the concentration of viable airborne bacteria or PM in the inlet (CFU/m^3^), T_c_ is the concentration of viable airborne bacteria or PM in treated air (CFU/m^3^).

#### 2.4.2. Particulate Matter Concentration Measurement

Real-time PM concentrations were measured by two DustTrak DRX Aerosol Monitors (#8533, TSI Inc., Shoreview, MN, USA) at five particulate size ranges (PM_1_, PM_2.5_, PM_4_, PM_10_, and TSP). Two aerosol monitors were used simultaneously for inlet and treated air, simultaneously with airborne bacteria sampling for 1 h (Figure S26). The sampling interval is 1 s, meaning that PM was measured and read by the monitors every second. The calculation of % PM mitigation shares the same method mentioned in Equation (9).

To quantify the pollutant removal efficiency of the FastAir prototype, CADR was calculated based on the following simplified Equation (7), according to Nelson et al. (1993) [29].

(10) CADR=ƞQ

where CADR is the clean air delivery rate (m^3^/s), ƞ is the single-pass particle removal efficiency of the device (between 0 and 1), and Q is the standard volumetric flow rate (m^3^/s) of the FastAir prototype.

### 2.5. Data Analysis

#### Statistical Analysis

For each treatment option (with *n* = 3 for experimental trials), the percentage (%) mitigation of viable airborne bacteria, PM, and sample standard deviations were calculated (Equation (6)). Statistical analysis was performed in Microsoft Excel and R studio (version 4.1.2). The one-sided Wilcoxon signed-rank test was conducted to analyze the statistical significance (*p*-value) comparing inlet and treated air results for airborne bacteria and PM concentrations.

In addition to the analysis of the data, normalization was also performed. The airborne bacteria concentration was normalized by simultaneously measuring PM concentrations at PM_1_ to TSP sizes, respectively, to determine how the PM size and morphology factor into the device performance.

(11) $$C_{norm}=\frac{C}{C_{PM}}$$

where *C_norm_* is the normalized concentration of viable airborne bacteria, *C* is the concentration of viable airborne bacteria in the sampled air (CFU/m^3^), *C_PM_* is the concentration of a certain PM concentration measured simultaneously with *C*. C_PM_ refers to all the PM categories measured in this experiment, from TSP, PM_10_, PM_4_, PM_2.5_, to PM_1_.

The normalization process used the viable airborne bacteria concentration (CFU/m^3^) divided by the concentration (µg/m^3^) of each PM size. The result is normalized airborne bacteria concentration per microgram of PM, and the yielding unit is CFU/µg. In this experimental environment (poultry demonstration/teaching room), the PM and viable airborne bacteria could not be controlled, and the concentrations varied at different times. It was assumed that higher PM concentrations generally tended to have higher airborne bacteria concentrations. The sizes of viable airborne bacteria are present in various PM sizes. However, the viable airborne bacteria measured and cultured in this experiment are mainly bacteria that contribute to the fine particulates measured in the PM_2.5_ and PM_1_ ranges. Therefore, the purpose of normalization was to deconvolute the performance of treatment options and elucidate additional performance data in the context of the measured PM sizes. This process can reduce the variability of viable airborne bacteria by scaling them with each PM concentration so as to find grounds for reasonable comparison and convenience of statistical analysis [30].

## 3. Results

### 3.1. Evaluation of the Actual Air Flow of the FastAir Prototype

As described in the previous section, the air flow rate of the FastAir prototype was measured using a FANS unit. The measured air flow rate was converted to the standard air flow based on Equations (5) and (6). The standard (defined as 20 °C, 1 atm, and dry air condition) air flow rates were summarized in Table 1, and the measured air flow rates were summarized in Table S1. The air flow rates were slightly reduced after the upgrade, but the reduction in air flow was not significant. Highlighted rows referred to the air flow rate of the intact FastAir prototype, with all parts installed during testing.

### 3.2. Mitigation of Airborne Pathogens

Table 2 summarizes the FastAir prototype’s effectiveness in removing airborne pathogen concentrations at percentage rates. All six treatment options in high and low flow rate modes were included. Regardless of the flow rate mode, the prototype mitigated more than 99% of viable airborne bacteria. It is also important to recognize that the “filtration” treatment mitigated over 98% of viable airborne bacteria in either flow rate mode. On the other hand, the “UV” treatment theoretically only mitigates viable airborne bacteria, and the efficacy depends on the dose: nearly 79% at the low dose (1.4 mJ/cm^2^, at the high flow rate) and nearly 95% at the high dose (2.9 mJ/cm^2^, at low flow rate). Taken together, it is assured to the user that the prototype has two ‘lines of defense’ against airborne pathogens (i.e., filtration and UV), which lowers the risk of accidental treatment failure.

A one-sided Wilcoxon signed-rank test was conducted to analyze the statistical significance between the inlet and treated air on airborne bacteria concentrations (Table 3) in *n* = 3 or 4 experimental trials. The inlet was blocked as all inlet samples (inlet air) in all six configuration options (Figure 5). Likewise, the treated air was blocked as all ‘treated air’ samples (outlet air) in all six configuration options (Figure 4). The calculated *p*-value was 1.063 × 10^−4^. A *p*-value that is less than 0.05 was assumed to indicate statistical significance. The calculated *p*-value is a conservative value because we treated all configurations in the same manner in statistical analysis, even though, for example, the “All” options performed better than the “UV only” options. This implied the actual quality of the prototype beyond what we tested.

### 3.3. Mitigation of Particulate Matter

Table 3 summarizes the removal efficiency of the FastAir prototype on airborne PM concentrations at percentage rates. All six configuration options were included. Regardless of flow rate mode, the prototype removed approximately 95% or more of TSP. The efficiency of PM removal decreased with the smaller PM size, i.e., from approximately 85%, 78%, and over 76% for PM_10_, PM_4_, and both PM_2.5_ and PM_1_, respectively. The “filtration only” configuration still removed approximately 97% or more of TSP, regardless of flow rate. There was a minor decrease in PM removal with decreased size (from 91% to 86%, but only for the high flow rate mode). The difference in PM removal efficiency was more apparent for finer PM compared with the TSP. This can be explained considering that TSP upper size range is ~100 µm, i.e., a much wider size range compared with, e.g., PM_10_ of ~10 µm, and a greater fraction of TSP is filtered out regardless of air flow rate.

Interestingly, the “UV” treatment removed PM (46% of TSP). However, that removal decreased to approximately 27% at the high flow rate for PM_10_ to PM_1_, and as low as 6% to 11% for a low flow rate. Much higher variability was also observed at the high flow rate. The most likely reason for this apparent ‘removal’ could be attributed to the Al mesh filter (Figure S5). It would not be reasonable to expect UV to treat PM (e.g., mineral dust), other than mitigating a relatively minor fraction of PM that is considered viable PM (i.e., viable airborne bacteria). In general, the “filtration only” option appeared to remove slightly more PM (up to 12% more for PM_1_) than the “all” option. However, this apparent difference is likely an artifact of different locations of air sampling ports, illustrated in Figure S15 (“filtration + UV”) and Figure S17 (“filtration only” configuration). The results were also within statistical random noise of each other (i.e., within the mean ± 2 sample standard deviations, for a 95% confidence interval).

Based on the results of Table 3, if we use TSP as the evaluation metric, the FastAir prototype removed 95% and 97% of TSP at high and low flow rates, respectively. We assumed that the particle removal efficiency (ƞ) in Equation (10) is the same as the two values above. Therefore, the CADR would be 0.95 m^3^/s and 0.48 m^3^/s for high and low flow rate modes, respectively.

### 3.4. Normalization of Airborne Pathogen Concentrations

Table 4 summarizes the efficiency of the FastAir prototype in removing normalized airborne bacteria concentrations (Equation (11)) on a percentage basis for all six configuration options under each PM normalized category. The normalization process used the airborne bacteria concentration (CFU/m^3^) divided by the simultaneously measured concentration (µg/m^3^) of each PM size, respectively. The result is normalized airborne bacteria concentration per microgram of PM. Unit: CFU/µg. Statistical analysis was conducted using the Wilcoxon signed-rank test. In the analysis, the comparison among different configuration options was not tested. However, all the *p*-values calculated were less than 0.05, indicating substantial statistical significance regarding the air-cleaning effects between the inlet and treated air.

The PM size and morphology influenced the prototype performance. Table 2 (pre-normalization) and Table 4 (post-normalization) can be compared to evaluate the normalization process. For H-F + UV and H-F with 100% bacteria reduction, normalized results remained the same. For H-UV and L-UV, the normalized percentage pathogen reduction with TSP was lower than other finer PM sizes. For the L-F “filtration only” option, due to the below detection limit for measured PM concentrations of the treated air in one of the trials, the normalization process could not be performed as the denominators were zero. For H-UV, L-F + UV, and L-F, they all have lower viable airborne bacteria percentage mitigation compared with pre-normalized data.

### 3.5. UV Dose Estimation

Most microbiology literature reports UV doses needed for the inactivation of pathogens using the same (energy/area) units. Therefore, it is appropriate to compare published data with the estimates in Table 5.

Table 5 summarizes the results of four methods for UV dose estimation mentioned in Material and Methods: Method 1: estimation by UV energy output; Method 2: single-plane estimation by measured irradiance; Method 3: multi-plane estimation by integration of measured irradiance. The results illustrate that the estimates are within an order of magnitude of each other, further highlighting the uncertainties related to UV treatment design.

Method 1, based on the nominal UV output, resulted in the highest estimated UV dose among the three methods. Methods 2 and 3 resulted in similar estimates on either 2-D or 3-D scale (within a 10% difference) of UV dose due to similar UV irradiance measurements.

Most UV-related literature record UV dose in term of energy per area (2-D). Therefore, only the 2-D UV dose estimated in this research can be used to compare with the literature. According to Sabino et al. (2020) [31], the UV-C (254 nm) lethal dose needed for surface-borne SARS-CoV-2 is 0.706 mJ/cm^2^ for 99% reduction and 6.556 mJ/cm^2^ for 99.9%. According to technical notes from ASHRAE (American Society of Heating, Refrigerating and Air-Conditioning Engineers) (ASHRAE, 2021) [32], the minimum dose for 90% inactivation of airborne SARS-CoV-2 is 0.611 mJ/cm^2^, and the extrapolated dose is 1.222 mJ/cm^2^ for 99% inactivation. ASHRAE recommended a conservative dose for 99% inactivation as 1.500 mJ/cm^2^ considering safety margins. Based on the more conservative (Method 3) UV dose on a 2-D scale (2.9 mJ/cm^2^ and 1.4 mJ/cm^2^, low and high flow rate mode, respectively), it can be assumed that it can sufficiently inactivate 99% of the airborne SARS-CoV-2 viruses under the low flow rate mode and nearly 99% for the high flow rate mode by UV-C light alone. This theoretical estimate addressed the research motivation to evaluate the applicability of the prototype to indoor air quality improvement in the post-COVID era.

## 4. Discussion

### 4.1. Testing Environment

The FastAir prototype was initially designed to be mounted on the ceilings of manufacturing floors where dust generation is high or in the auditorium, theaters, and public areas where people congregate. The prototype was not meant to be used on livestock farms. However, for testing purposes, poultry farms tend to have much higher PM and airborne bacteria concentrations than normal human residential or workspaces, and therefore the mitigation effect can easily be compared between the inlet and treated air. Regarding the percentage (%) mitigation we used in this paper, it is essential to note that testing in a clean environment may eventually achieve 100% mitigation of viable airborne bacteria and PM, but the effectiveness cannot be verified because the “baseline” (inlet air) is too low to reliably detect. Therefore, testing in an environment with a higher “background” (inlet) level enables a more realistic assessment of the removal efficiency of the prototype and, therefore, higher confidence in reported performance.

### 4.2. Airborne Pathogen and PM Mitigation

For viable airborne bacteria, both “filtration only” and “filtration + UV” options achieved up to 100% reduction for both high and low flow rate modes. The “UV only” treatment achieved a higher effect in the low flow rate mode than in the high flow rate mode. This is likely due to the higher UV dose resulting from a longer treatment time. The “filtration + UV)” option is recommended for maximum protection from viable airborne bacteria. However, the “filtration only” treatment can also be effective in less critical applications. For PM concentrations, both “filtration only” and “filtration + UV” options achieved more than 94% for both modes for total PM. The low flow rate mode performed better than the high flow rate mode. The “UV only” option (with Al mesh filters) obtained much lower reduction rates, especially at a high flow rate. That was because Al mesh filters have very coarse pore sizes compared to MERV-8 or MERV-13; they were not expected to filter PM effectively. The purpose of the Al mesh filters was to block most of the UV irradiation on MERV-13 filters to extend the lifespan of filtration.

### 4.3. Data Analysis

A one-sided Wilcoxon signed-rank test was chosen as an alternative to the t-test. The t-test requires normality or a large sample size, which we had neither in his case. The Wilcoxon test ranks the observations from lowest to highest and performs the test on these ranks. It tested a more general hypothesis, i.e., is the probability of the airborne pathogen concentrations in the inlet group higher than in the treated groups?

Lee et al. (2021) [30] showed increased statistical significance after normalizing airborne pathogen concentration by PM concentrations. However, in our study, the statistical significance was powerful without normalization, but the normalization was performed to elucidate more data about the prototype performance.

### 4.4. Potential Improvement

This study tested the air treatment effect of the Fast Air prototype (1 h simultaneous sampling of inlet and treated air samples). The results showed significant removal efficiency of both PM and viable airborne bacteria; however, the more prolonged treatment effect regarding the room air quality is forthcoming in a companion manuscript.

Due to the original structure of the prototype built by the company, we did not make changes to the blower or the motor itself. In environments with a higher flow rate, more powerful motors and blowers could be installed to satisfy the need. On the other hand, this prototype can be modular, i.e., users can stack several units in parallel to achieve the target air flow rate or air change rate or increase the prototype size. Another improvement that could be made is to have the lamp fixtures mounted on the inner side walls rather than attached to the lamps. That would require more labor work on installation, but it would enhance the germicidal ability of UV as UV light can emit omnidirectionally without obstruction. Some safety enhancements, such as lamp clamps on UV bulbs, would reduce the risk of bulbs falling and breaking during moving and other accidents.

As shown in Figure 2, the sampling ports and tubes for the treated air were perpendicular to the air flow direction in the FastAir prototype. Therefore, it was regarded as misalignment sampling described by Zhang (2000) [33], which made the sampling efficiency lower than when the sample tubes were parallel to the sampling air flow due to the inertia effect. In addition, the mean air sampling velocity in the PTFE tubes was much higher than the average air velocity inside the FastAir prototype in low flow rate mode and similar to that in high flow rate mode; therefore, the sampling could be regarded as superisokinetic sampling in low flow rate mode, which also made the sampling efficiency lower. Adjustments can be made in future scenarios to improve the sampling efficiency to facilitate isokinetic sampling: sampling tubes can be positioned in parallel with the air flow inside the FastAir prototype; sampling velocity can be adjusted lower to be consistent with the air flow rate inside the FastAir prototype.

Desiccation of sampling fluid was noted during each 1 h sampling experiment. The liquid in each BioSampler^®^ lost over 5 mL on average during the impingement process. That observation was consistent with some prior studies where desiccation of BioSampler^®^ liquid was observed but not significant. Due to the desiccation effect, extensive sampling with BioSamplers^®^ might not be suitable. Alternative sampling methods may be needed if a more extended sampling method is needed.

### 4.5. Safety Concern on Ozone

It is well known that ozone generation by UV light strongly depends on the wavelength. Ozone generation efficiently occurs with UV below 240 nm, due to trend of UV absorption by oxygen. The maximum efficacy of ozone generation by UV happens at approximately 160 nm. On the other hand, the absorption (mitigation) of ozone is also related to UV wavelength. The peak ozone absorption happens at about 254 nm. Therefore, theoretically, low-pressure mercury UV-C bulbs at 254 nm generate minimal amounts of ozone and can also mitigate ozone simultaneously [34]. Note that the UV bulb used in this experiment has a peak UV wavelength of 254 nm with minimal output at other UV wavelengths [19].

## 5. Conclusions

The FastAir prototype was equipped with germicidal UV-C lamps with enhanced mitigation capabilities against viable airborne bacteria. The FastAir prototype performance was tested at an indoor air location characterized by higher dust and pathogen levels than in regular residential areas and the pollutant removal efficiency of the upgraded FastAir prototype was validated for broader applications.

The low flow rate mode provided a better overall treatment effect than the high flow rate for both PM and airborne pathogen concentrations. The “filtration + UV” option is recommended for maximum protection from viable airborne bacteria. However, the “filtration only” configuration can also be effective in less critical applications.

The prototype (“filtration + UV”) mitigated more than 99% of viable airborne bacteria between the inlet and treated air regardless of the flow rate mode. The “filtration only” configuration mitigated over 98% of viable airborne bacteria in either flow rate mode. The “UV only” configurations mitigated nearly 79% at the low dose (high flow rate mode) and nearly 95% at the high dose (low flow rate mode).

Regardless of the flow rate mode, the prototype removed 95% or more of TSP. The efficacy of PM removal decreased with the lower PM size. The “filtration only” configuration removed 97% or more of TSP, regardless of flow rate. The “UV only” option (UV light + Al mesh filters) removed 46% or more of TSP.

Normalization of airborne bacteria concentrations by PM concentrations was conducted as an additional effort to help us better understand the data. For the “UV only” configuration, the normalized bacteria reduction first increases and then decreases with decreasing PM sizes.

## Figures and Tables

**Table 1:** Summary of air flow rate (converted to standard air flow rate) of the FastAir device under different conditions. Bold values refer to the conditions that test the performance of the entire prototype.

Prototype	Flow Rate Mode	Conditions (Load)	Standard Air Flow Rate *	
			m^3^/s	CFM **
**Original**	High	No filters	1.28	2704
		MERV 13 only	1.08	2280
		**MERV 8 & 13**	**1.01**	**2146**
	Low	No filters	0.86	1813
		MERV 13 only	0.61	1283
		**MERV 8 & 13**	**0.57**	**1200**
**Upgraded with UV**	High	UV light only	1.20	2537
		UV light + Al mesh	1.16	2440
		UV light + Al mesh + MERV 13	1.02	2154
		**UV light + Al mesh + MERV 8 & 13**	**1.00**	**2101**
	Low	UV light only	0.53	1115
		UV light + Al mesh	0.51	1076
		UV light + Al mesh + MERV 13	0.49	1037
		**UV light + Al mesh + MERV 8 & 13**	**0.49**	**1027**

* Standard air flow conditions are defined as 20 °C, 1 atm, and dry air condition. ** CFM: cubic feet per cubic meter, a customary unit commonly used in the ventilation industry in the United States.

**Table 2:** Summary of the removal of viable airborne bacteria from the air. The performance metric is the percent reduction defined as the relative difference between inlet and treated concentrations under different configuration options. The percentage is the average of three or four trials conducted for each configuration. The loading of average airborne bacteria concentrations in the inlet air of each trial varied from 444 CFU/m^3^ to 7378 CFU/m^3^.

Configuration Option	Mean Percentage Mitigation of Airborne Bacteria Concentrations ± SD *
H-F + UV	100%
H-F	100%
H-UV	79% ± 5%
L-F + UV	99% ± 2%
L-F	98% ± 3%
L-UV	95% ± 5%

* SD: sample standard deviation. The quoted sample standard deviations are based on the data from the *n* = 3 or 4 trials. In some instances, identical values were obtained in all measurements and no sample standard deviation is cited.

**Table 3:** The mean percentage mitigation of PM concentrations between the inlet and treated air under different configuration options. The performance metric is the percent reduction defined as the relative difference between inlet and treated concentrations under different configuration options. The percentage is the average of three or four trials conducted for each configuration. The loading of average TSP in the inlet air of each trial varied from 97 µg/m^3^ to 230 µg/m^3^.

Configuration Option	Mean Percentage of PM Removed (%R) ± SD *				
	TSP	PM_10_	PM_4_	PM_2.5_	PM_1_
H-F + UV	95% ± 2%	85% ± 5%	78% ± 7%	77% ± 8%	76% ± 8%
H-F	97% ± **%	91% ± 1%	87% ± 3%	87% ± 3%	86% ± 3%
H-UV	50% ± 25%	27% ± 24%	27% ± 17%	30% ± 19%	30% ± 19%
L-F + UV	97% ± 3%	91% ± 6%	87% ± 8%	87% ± 9%	88% ± 9%
L-F	100% ± **%	100% ± **%	100% ± **%	100% ± **%	100% ± **%
L-UV	46% ± 5%	11% ± 2%	6% ± 2%	7% ± 2%	7% ± 1%

* SD: sample standard deviation. ** Apparent sample standard deviation <1%.

**Table 4:** The percentage removal of viable airborne bacteria is normalized by different PM sizes.

Configuration Options	Normalized Percentage of Airborne Bacteria Mitigation (%R)				
	by TSP	by PM_10_	by PM_4_	by PM_2.5_	by PM_1_
H-F + UV	100%	100%	100%	100%	100%
H-F	100%	100%	100%	100%	100%
H-UV	54% ± 9%	71% ± 2%	71% ± 1%	70% ± 1%	70% ± 1%
L-F + UV	68% ± 56%	90% ± 17%	93% ± 12%	94% ± 11%	94% ± 11%
L-F	N/A *	N/A	N/A	N/A	N/A
L-UV	91% ± 7%	94% ± 4%	95% ± 4%	95% ± 4%	94% ± 4%
*p*-Values	0.0171	0.0005	0.0003	0.0003	0.0006

* N/A refers to the fact that PM concentrations for some of the treated air samples were recorded below the detection limit for one trial, so the normalization calculation is not viable (denominators were zero). The quoted sample standard deviations are based on the data from the *n* = 3 or 4 trials. In some instances, identical values were obtained in all measurements and no sample standard deviation is cited.

**Table 5:** Summary of UV dose estimations based on the three methods.

UV Dose		Method 1		Method 2		Method 3	
		2-D (mJ/cm^2^)	3-D (mJ/cm^3^)	2-D (mJ/cm^2^)	3-D (mJ/cm^3^)	2-D (mJ/cm^2^)	3-D (mJ/cm^3^)
Flow Rate Mode	High	6.3	0.45	1.5	0.22	1.4	0.22
	Low	12.8	0.92	3.1	0.11	2.9	0.11

## Data Availability

Please refer to the Supplementary Material for additional information and data about this research.

## Supplementary Materials

The following supporting information can be downloaded at: https://www.mdpi.com/article/10.3390/ijerph192316135/s1, Figure S1. The original FastAir prototype (prior to modification) with side views. The outer filters are MERV-8, and the inner filters are MERV-13. Figure S2. Sideview of the original FastAir prototype (prior to upgrade and modification) with side views. The outer filters are MERV-8 filters (left), and the inner filters are MERV-13 fiberglass pocket filters (right). Figure S3. Half-side view of the previous configuration (top figure): MERV-8 and MERV-15 filters were stacked in series. Figure S4. Half-side view of the upgraded configuration (bottom figure): the 15-in deep MERV-13 filters were replaced by 4-in deep MERV-13 filters, 2-in deep Al mesh filters, and UV-C lamps. Figure S5. Aluminum mesh filters were installed between UV light and MERV filters to allow sufficient air flow while blocking most of the UV light from irradiating on MERV filters. Air sampling tubes were omitted in this photo. Figure S6. A close view of the controller box that controls the motor/blower and both sides of UV light. Figure S7. Schematic of UV irradiance measurements in cross-sectional planes that are parallel to UV lamps for UV dose estimation. Irradiance was measured at the 7 × 5 grids. Figure S8. The summary of UV detector measurement results from 2.5 cm (1 in) to 13 cm (5 in) away from the UV lamp. Each data point represents an average UV irradiance measured at a distance from the lamps’ plane on a virtual cross-sectional plane parallel to the lamp on a 7 × 5-point grid. The results are somewhat contrary to intuition because the measured irradiance does not reach its maximum at the nearest distance (2.5 cm), but at around 7.6 cm away from the UV light. Figure S9. The FastAir prototype was connected to a 3.0 m foam duct, and its larger end was connected to the FANS prototype for air flow measurement. Figure S10. Cross-sectional photo of air measurement duct. The photo was taken from the larger opening to the smaller opening (which was connected to the FastAir prototype). Figure S11. A caged poultry room with about 150 laying hens was selected for testing the FastAir prototype. Figure S12. Testing of FastAir prototype overall performance in a high air flow mode with simultaneous Filtration and UV treatments. Figure S13. Testing of FastAir prototype’s UV treatment performance in a high air flow mode. Filtration was removed. Figure S14. Testing of FastAir prototype’s Filtration treatment performance in a high air flow mode. The UV light was off. Figure S15. Testing of FastAir prototype overall performance in a low air flow mode with simultaneous filtration and UV treatments. Figure S16. Testing of FastAir prototype’s UV treatment performance on a high air flow mode. Filtration was removed. Figure S17. Testing of FastAir prototype’s Filtration treatment performance in a high air flow mode. The UV light was off. Figure S18. Sampling ports and tubes immediately after MERV-13 filters and before aluminum mesh filters. The left one (white color) was used to sample viable airborne bacteria, and the right one (transparent) was used to sample PM. Figure S19. Closeup of sampling ports connected with tubing. Left: the port connected with a Tygon tubing (ID = 9.5 mm or 3/8 in) that was used for PM sampling; right: the port was connected with a hard PTFE (polytetrafluoroethylene) tubing (ID = 9.5 mm or 3/8 in, OD = 12.7 mm or ½ in) that was used for airborne pathogen sampling. Other ports that were not used at the moment were closed and sealed with caps. Figure S20. A closeup view of sampling ports inside the FastAir prototype. The ports can be connected to PTFE tubing or Tygon tubing for air sampling. The tubing was omitted in this photo. Figure S21. MERV-13 filters were installed right after MERV-8 filters as the 2nd layer to be in contact with the inlet air. Sampling ports and tubes can be seen in this photo. The left one (white color) was for sampling viable airborne bacteria, and the right one (transparent) was for sampling PM. Figure S22. Details of airborne bacteria sampling using SKC BioSamplers^®^. The BioSamplers^®^ were placed in ice during the sampling process to ensure the viability of the microbes captured. Figure S23. Details of sampling aerosols on a cart house two sampling stations. The left one (treated air) was connected to the ports on top of the FastAir unit, while the right one (inlet air) was for sampling the aerosols in the room. Each station is connected to a vacuum pump on the bottom of the shelf of the cart. A total of six BioSamplers^®^ can be seen in the photo, three per sampling station. Figure S24. Two vacuum pumps (one for the inlet and one for treated air) were used to power the air sampling through the BioSamplers^®^. Figure S25. The results of incubation after 24 h of inoculation of the BioSampler^®^ fluid. The three left plates showed aerobic growth (inlet), while the three right plates showed no aerobic growth (treated air). The final plate counts started after 48 h of incubation. Figure S26. Details of PM measurements. Two DustTrak monitors (blue) are shown in the photo. The closer (left) one connects with both sides of the sampling ports on top of the FastAir prototype immediately after MERV-8 filters (treated air). The farther (right) one directly measures the PM concentrations in the air (inlet). Table S1. Summary of air flow rate measured by the FANS unit of the FastAir device under different conditions. These raw air flow rates were converted to standard air flow rates, and the results can be seen in Table 1 in the main text. Table S2. Testing of the FastAir prototype in a clean laboratory environment. Simultaneous sampling of Control (*n* = 3) and Treatment (*n* = 3) was conducted for airborne pathogens for 1 h. The results are expressed in CFU/mL. Table S3. Testing of the FastAir prototype in a clean laboratory environment. Real-time PM concentrations were measured by DustTrak monitors on the inlet and treated air for 1 h, simultaneously with the airborne bacteria sampling mentioned in Supplementary Table S1. The PM concentrations are expressed in µg/m^3^.
