# Supplementary material for: Evaluation of an Air Cleaning Device Equipped with Filtration and UV: Comparison of Removal Efficiency on Particulate Matter and Viable Airborne Bacteria in the Inlet and Treated Air
Source: Int J Environ Res Public Health. 2022 Dec 2;19(23):16135. doi: 10.3390/ijerph192316135 (PMC9735963; doi:10.3390/ijerph192316135)
Supplement: Supplementary file 1 [file ijerph-19-16135-s001.zip › ijerph-2046178-supplementary.pdf]

International Journal of
Environmental Research and Public Health

MDPI

Supplementary Material

Evaluation of an air cleaning device equipped with filtration and UV: comparison of removal efficiency on particulate matter and viable airborne bacteria in the inlet and treated air

Li et al. (2022)

1. Supplementary Figures

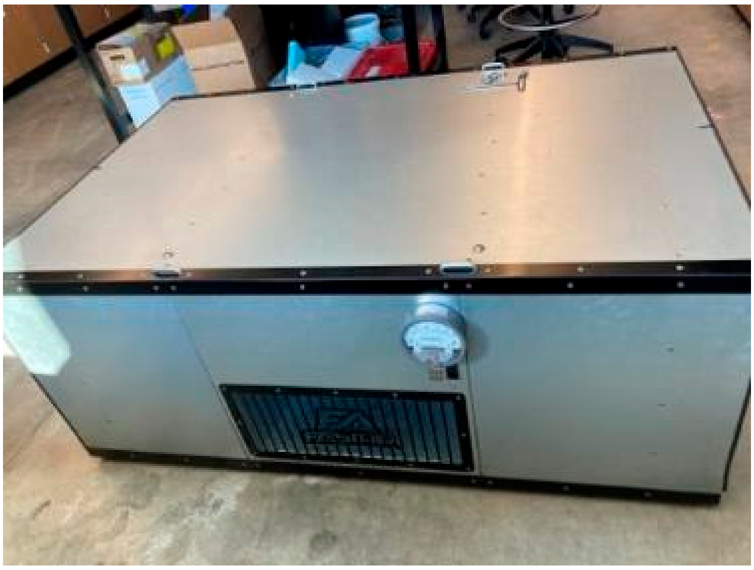

Figure S1. The original FastAir prototype (prior to modification) with side views. The outer filters are MERV-8, and the inner filters are MERV-13.

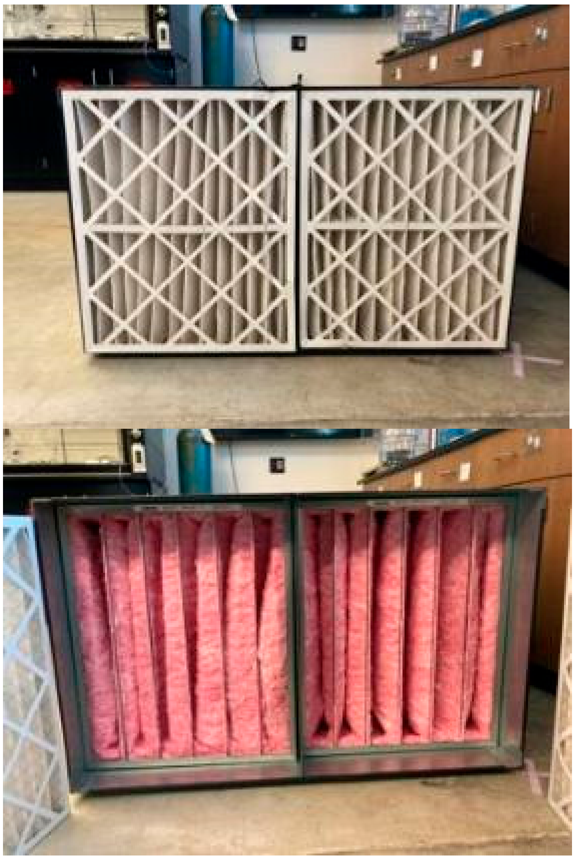

Figure S2. Sideview of the original FastAir prototype (prior to upgrade and modification) with side views. The outer filters are MERV-8 filters (left), and the inner filters are MERV-13 fiberglass pocket filters (right).

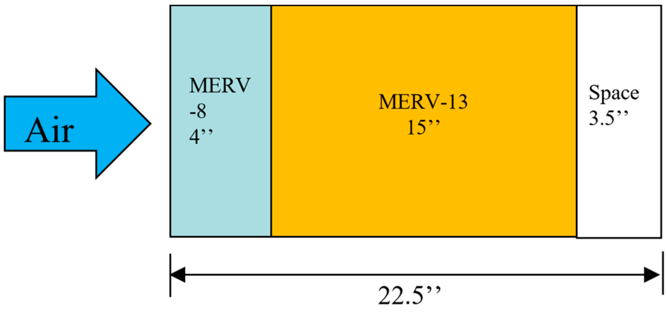

Figure S3. Half-side view of the previous configuration (top figure): MERV-8 and MERV-15 filters were stacked in series.

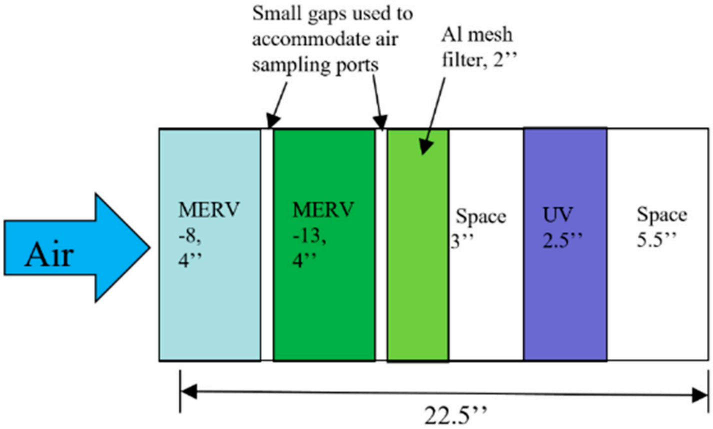

Figure S4. Half-side view of the upgraded configuration (bottom figure): the 15-in deep MERV-13 filters were replaced by 4-in deep MERV-13 filters, 2-in deep Al mesh filters, and UV-C lamps.

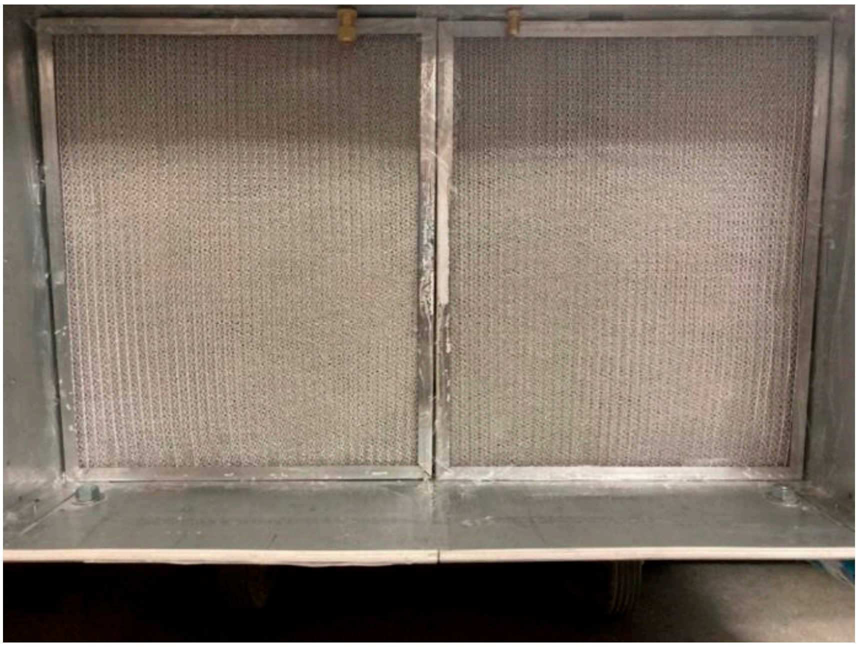

Figure S5. Aluminum mesh filters were installed between UV light and MERV filters to allow sufficient air flow while blocking most of the UV light from irradiating on MERV filters. Air sampling tubes were omitted in this photo.

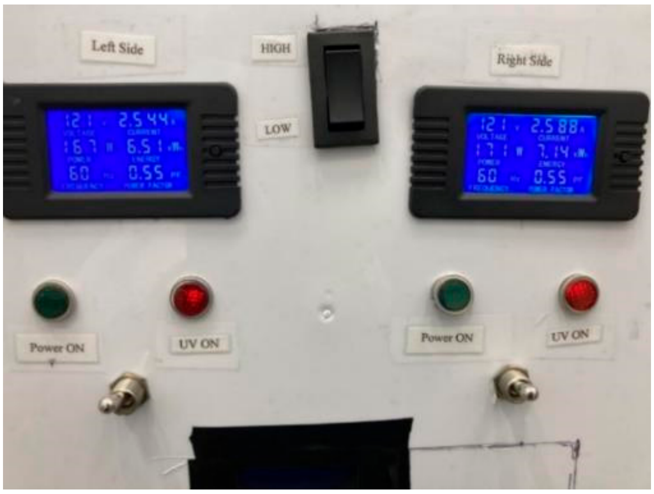

Figure S6. A close view of the controller box that controls the motor/blower and both sides of UV light.

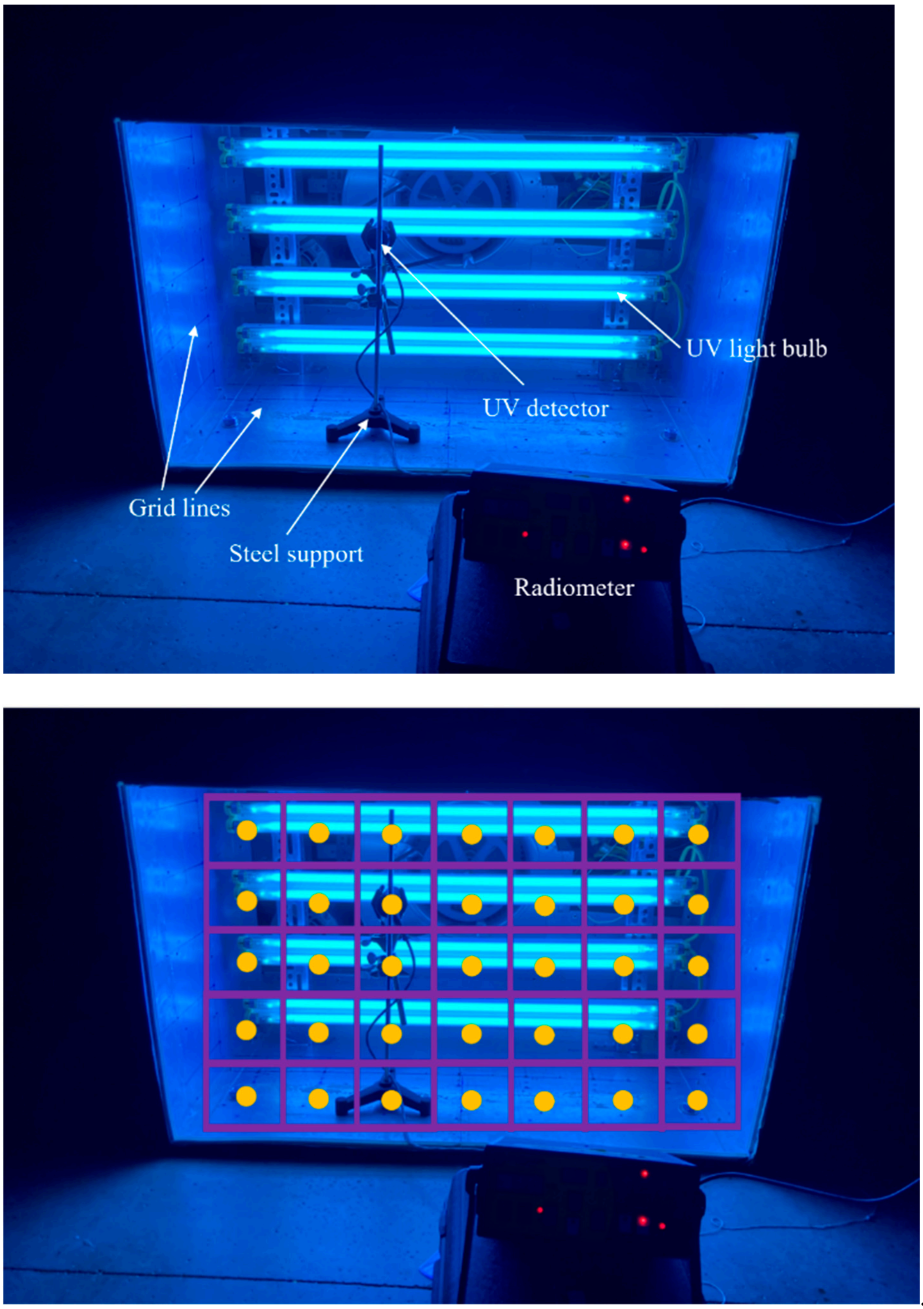

Figure S7. Schematic of UV irradiance measurements in cross-sectional planes that are parallel to UV lamps for UV dose estimation. Irradiance was measured at the 7 × 5 grids.

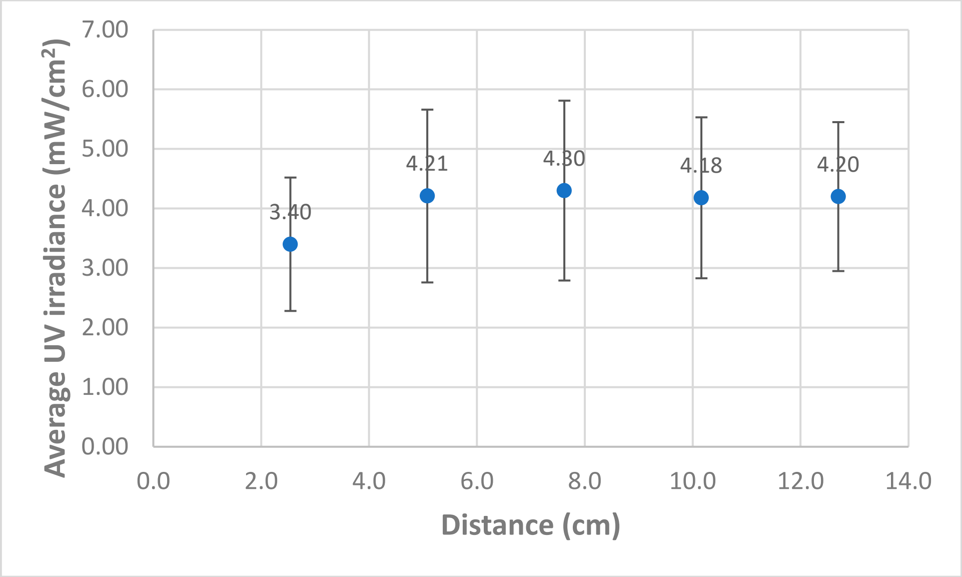

Figure S8. The summary of UV detector measurement results from 2.5 cm (1 in) to 13 cm (5 in) away from the UV lamp. Each data point represents an average UV irradiance measured at a distance from the lamps' plane on a virtual cross-sectional plane parallel to the lamp on a 7×5-point grid. The results are somewhat contrary to intuition because the measured irradiance does not reach its maximum at the nearest distance (2.5 cm) but at around 7.6 cm away from the UV light.

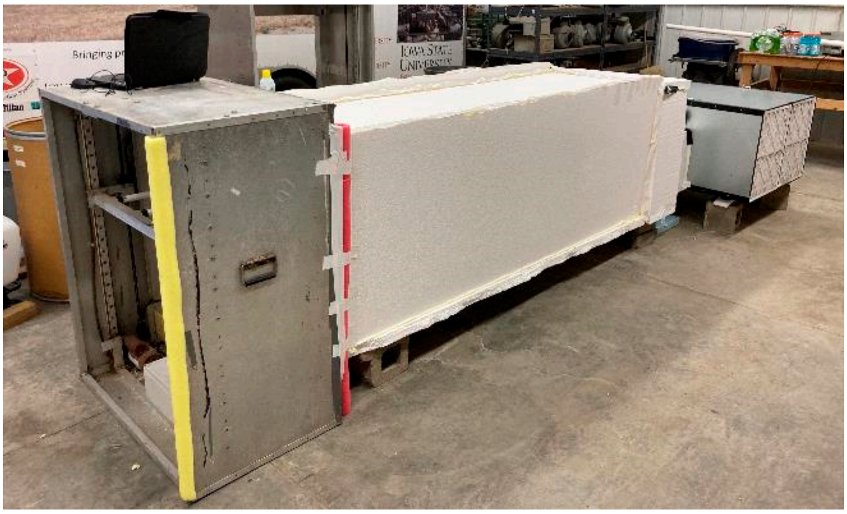

Figure S9. The FastAir prototype was connected to a 3.0 m foam duct, and its larger end was connected to the FANS prototype for air flow measurement.

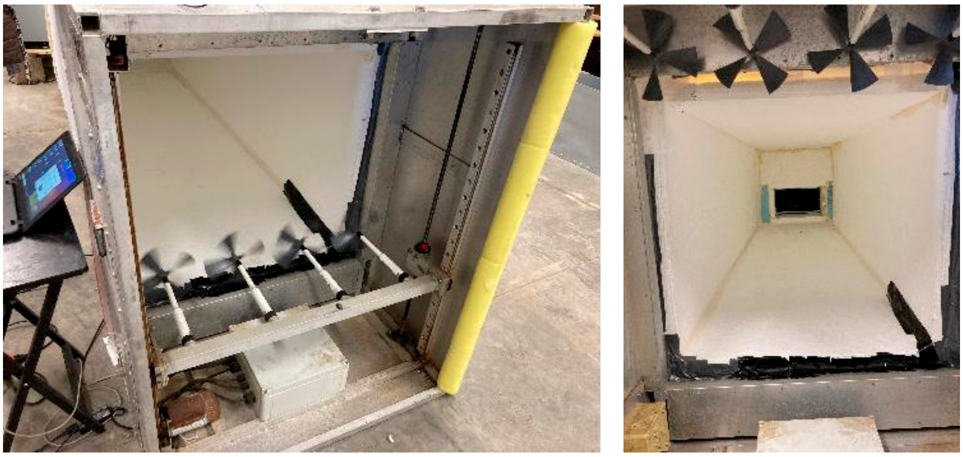

Figure S10. Cross-sectional photo of air measurement duct. The photo was taken from the larger opening to the smaller opening (which was connected to the FastAir prototype).

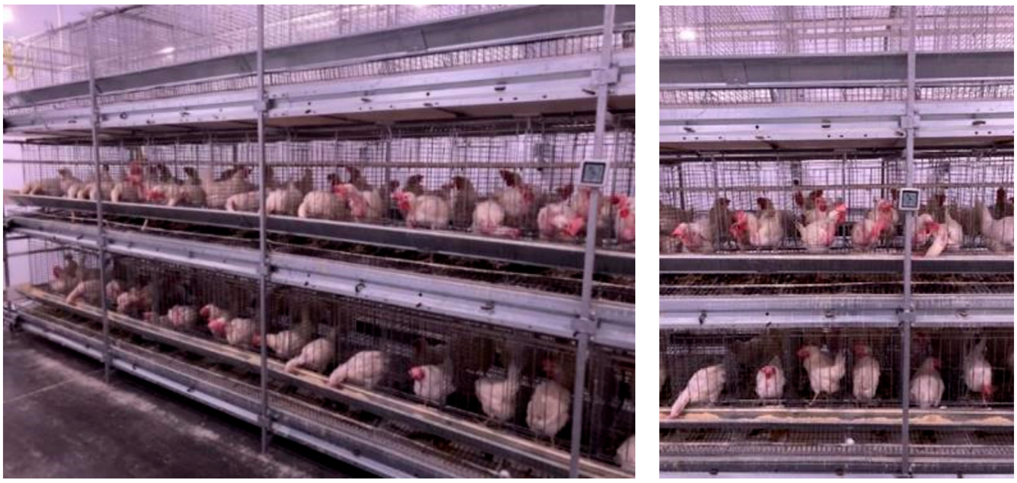

Figure S11. A caged poultry room with about 150 laying hens was selected for testing the FastAir prototype.

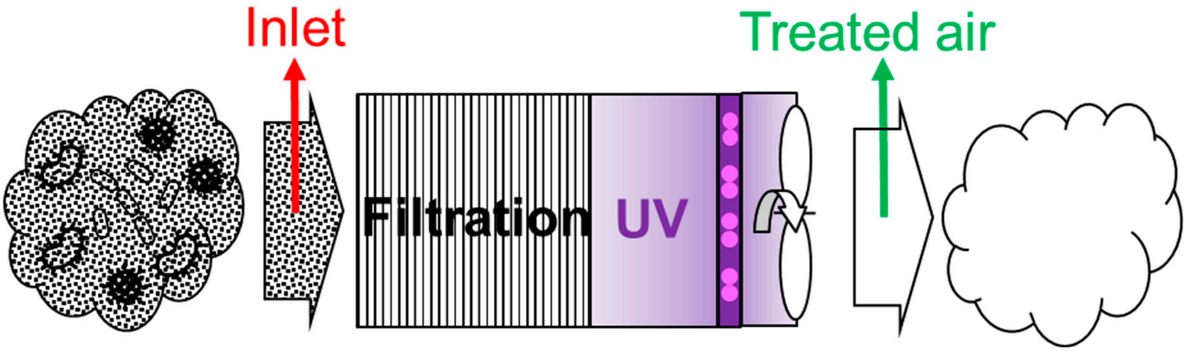

Figure S12. Testing of FastAir prototype overall performance in a high air flow mode with simultaneous Filtration and UV treatments. .

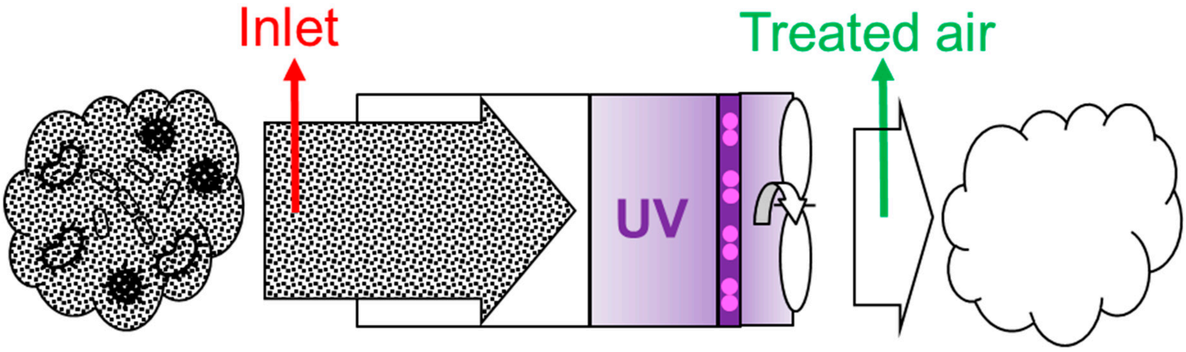

Figure S13. Testing of FastAir prototype's UV treatment performance in a high air flow mode. Filtration was removed. .

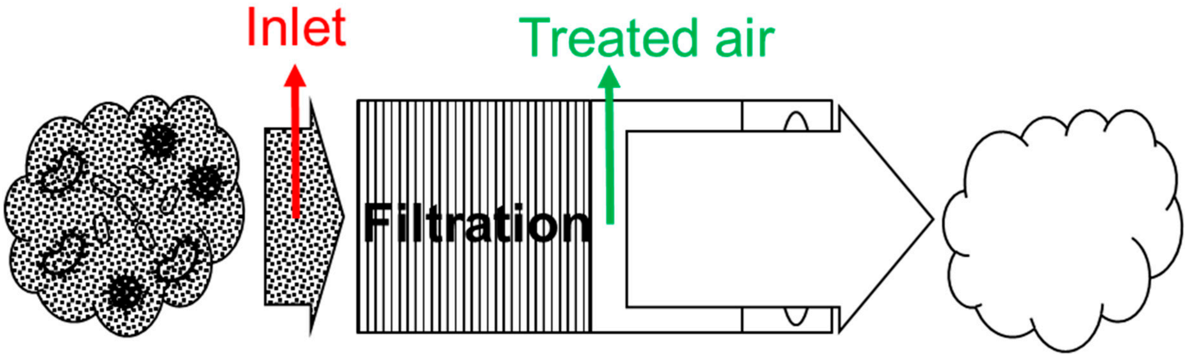

Figure S14. Testing of FastAir prototype's Filtration treatment performance in a high air flow mode. The UV light was off. .

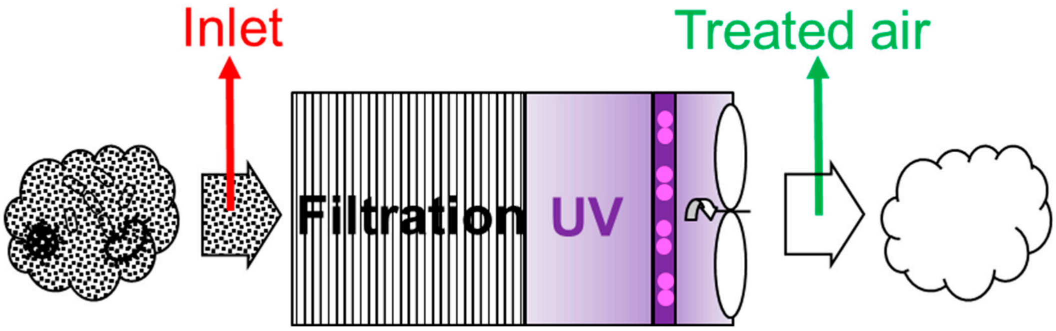

Figure S15. Testing of FastAir prototype overall performance in a low air flow mode with simultaneous filtration and UV treatments. .

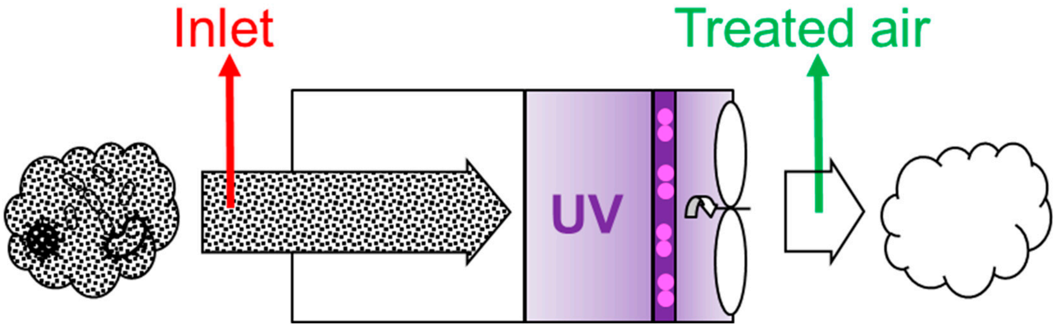

Figure S16. Testing of FastAir prototype's UV treatment performance on a high air flow mode. Filtration was removed. .

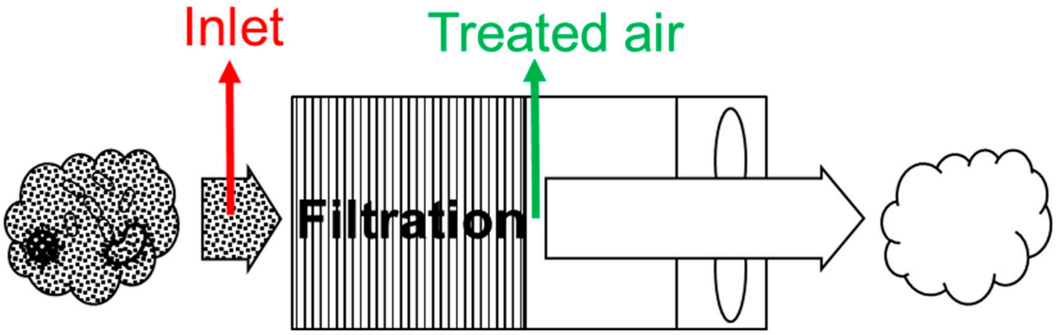

Figure S17. Testing of FastAir prototype's Filtration treatment performance in a high air flow mode. The UV light was off. .

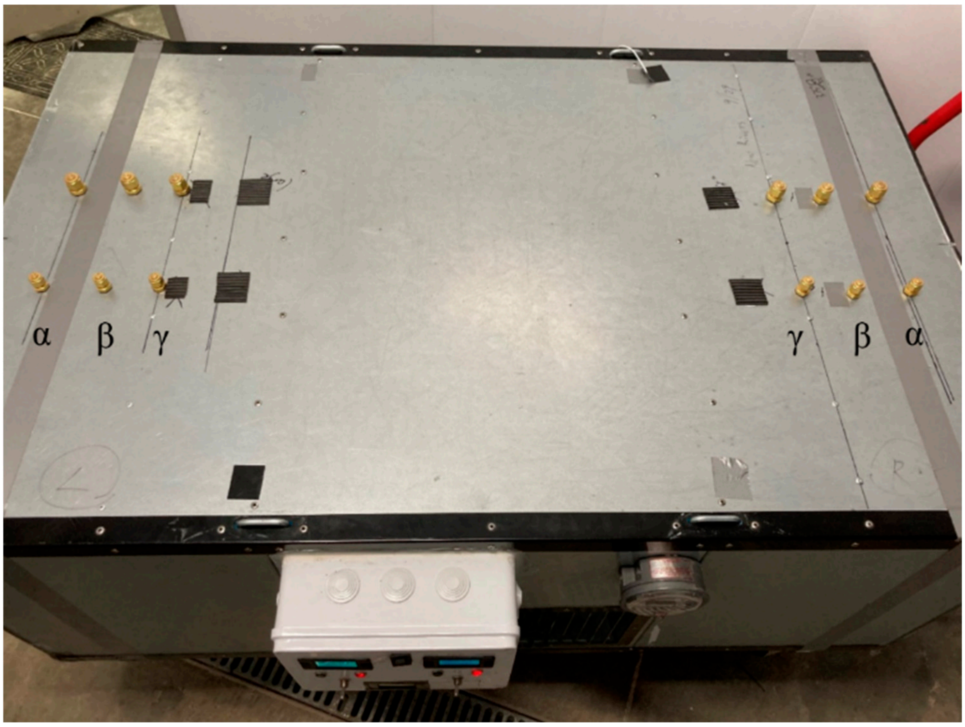

Figure S18. Sampling ports and tubes immediately after MERV-13 filters and before aluminum mesh filters. The left one (white color) was used to sample viable airborne bacteria, and the right one (transparent) was used to sample PM.

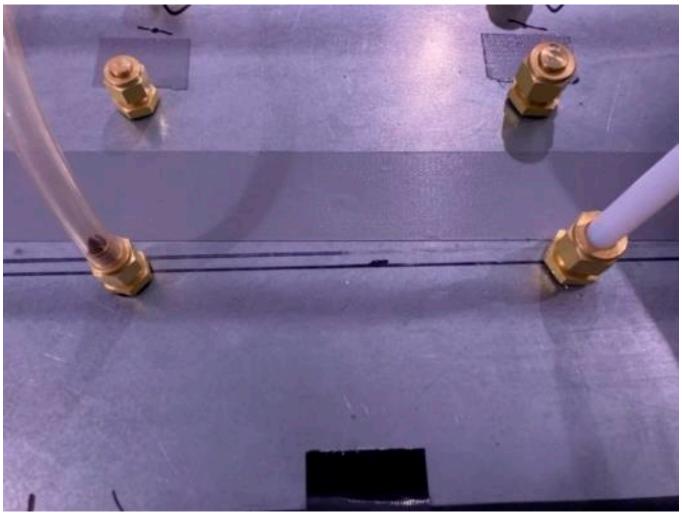

Figure S19. Closeup of sampling ports connected with tubing. Left: the port connected with a Tygon tubing (ID = 9.5-mm or 3/8-in) that was used for PM sampling; Right: the port was connected with a hard PTFE (polytetrafluoroethylene) tubing (ID = 9.5-mm or 3/8-in, OD = 12.7-mm or ½-in) that was used for airborne pathogen sampling. Other ports that were not used at the moment were closed and sealed with caps.

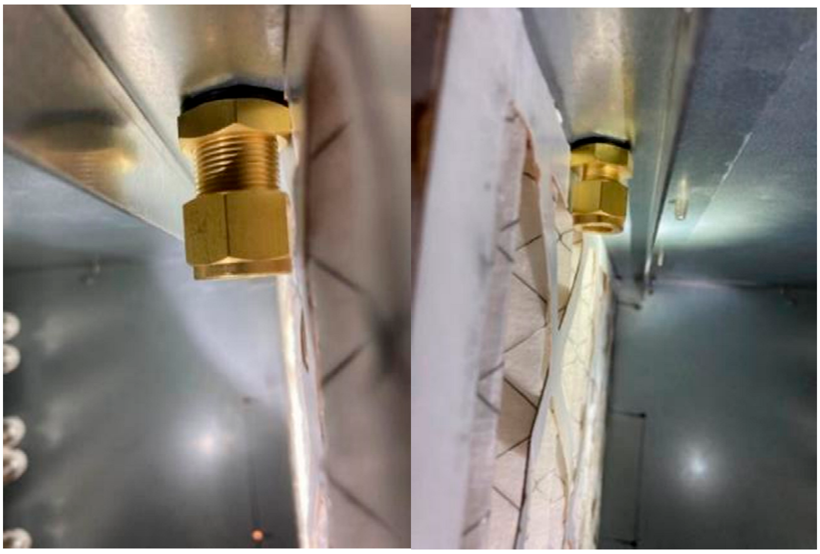

Figure S20. A closeup view of sampling ports inside the FastAir prototype. The ports can be connected to PTFE tubing or Tygon tubing for air sampling. The tubing was omitted in this photo.

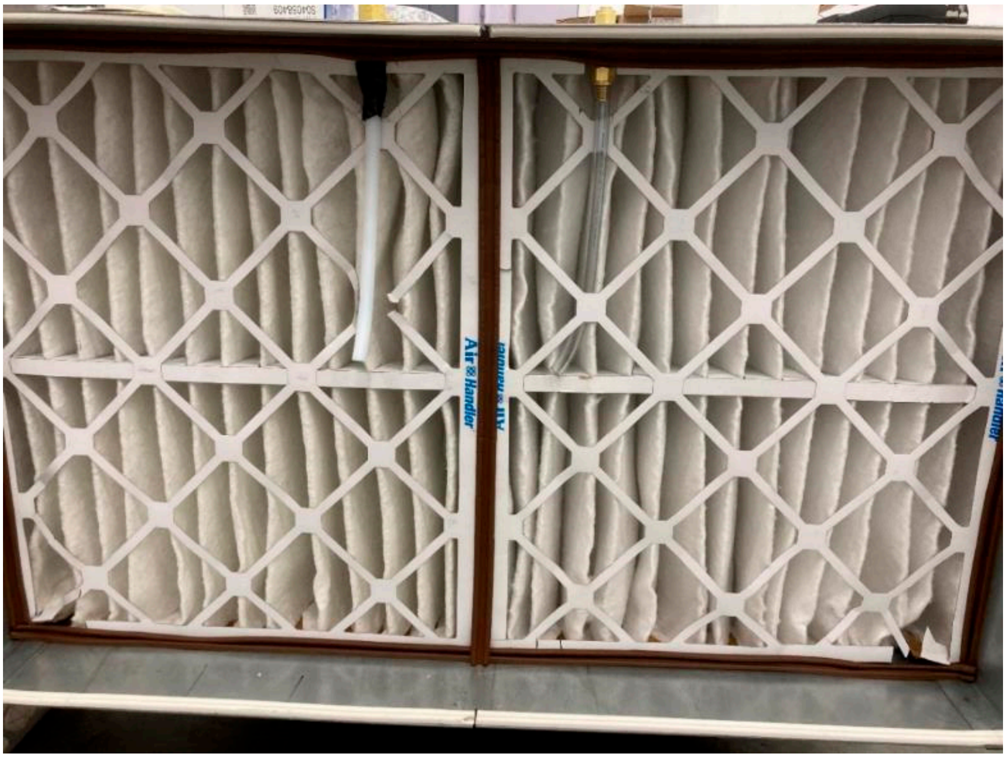

Figure S21. MERV-13 filters were installed right after MERV-8 filters as the 2nd layer to be in contact with the inlet air. Sampling ports and tubes can be seen in this photo. The left one (white color) was for sampling viable airborne bacteria, and the right one (transparent) was for sampling PM.

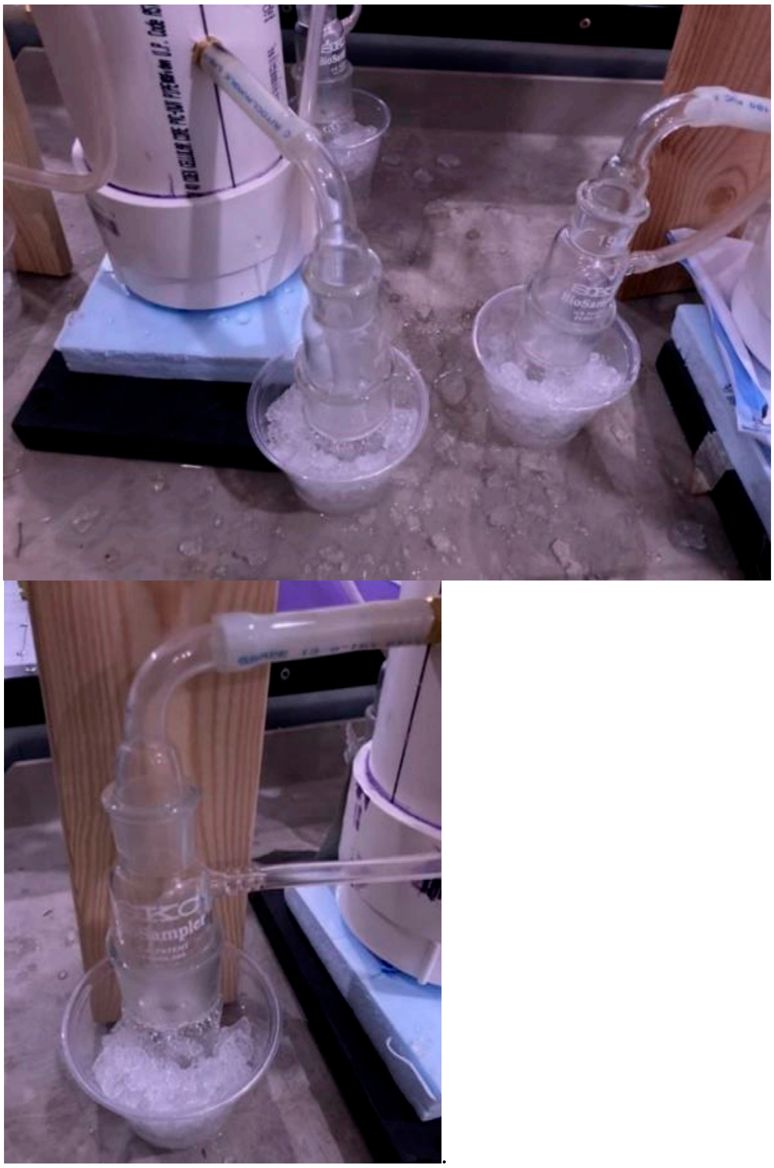

Figure S22. Details of airborne bacteria sampling using SKC BioSamplers®. The BioSamplers® were placed in ice during the sampling process to ensure the viability of the microbes captured.

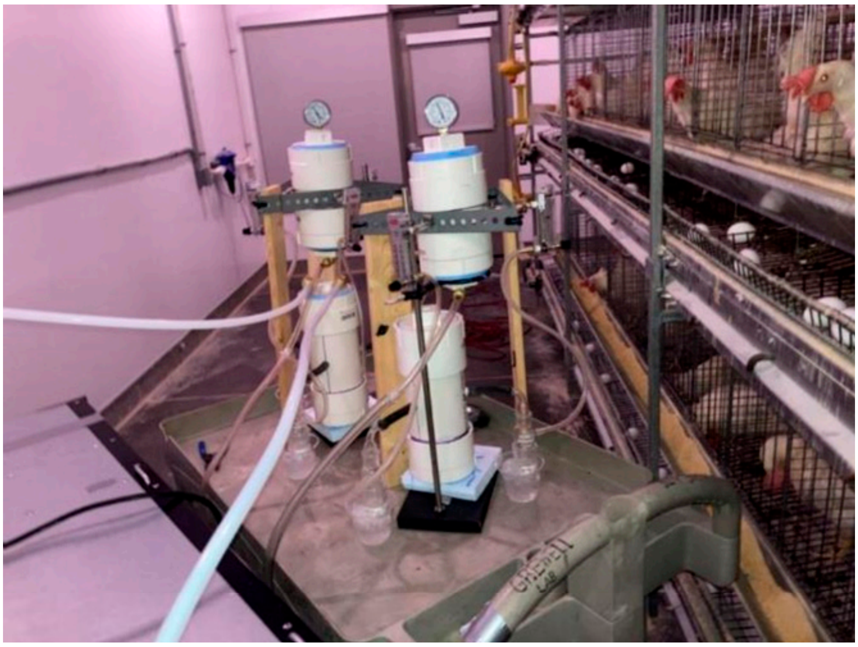

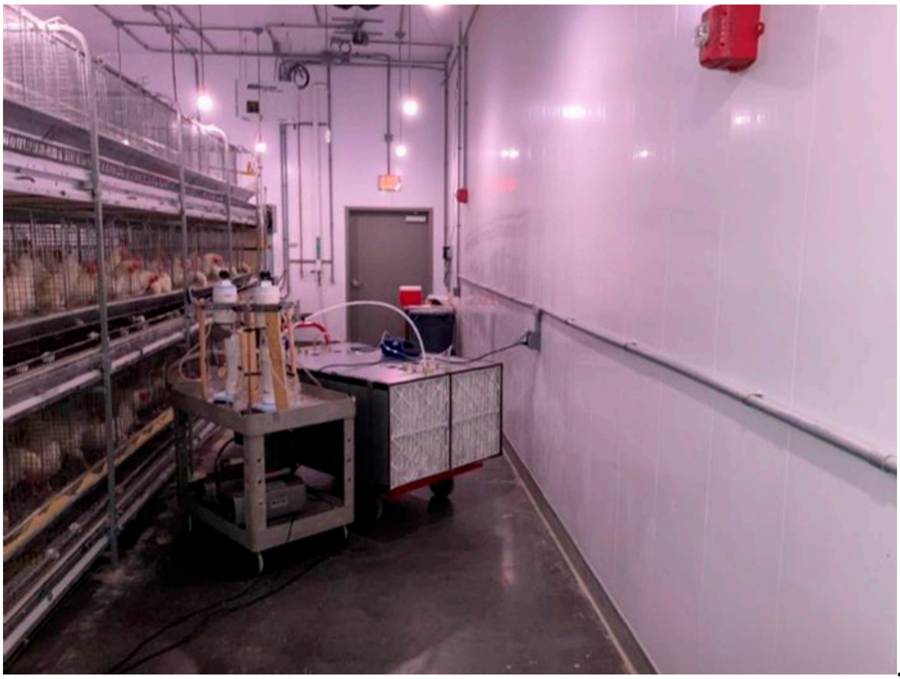
.

Figure S23. Details of sampling aerosols on a cart house two sampling stations. The left one (treated air) was connected to the ports on top of the FastAir unit, while the right one (inlet air) was for sampling the aerosols in the room. Each station is connected to a vacuum pump on the bottom of the shelf of the cart. A total of 6 BioSamplers® can be seen in the photo, 3 per sampling station.

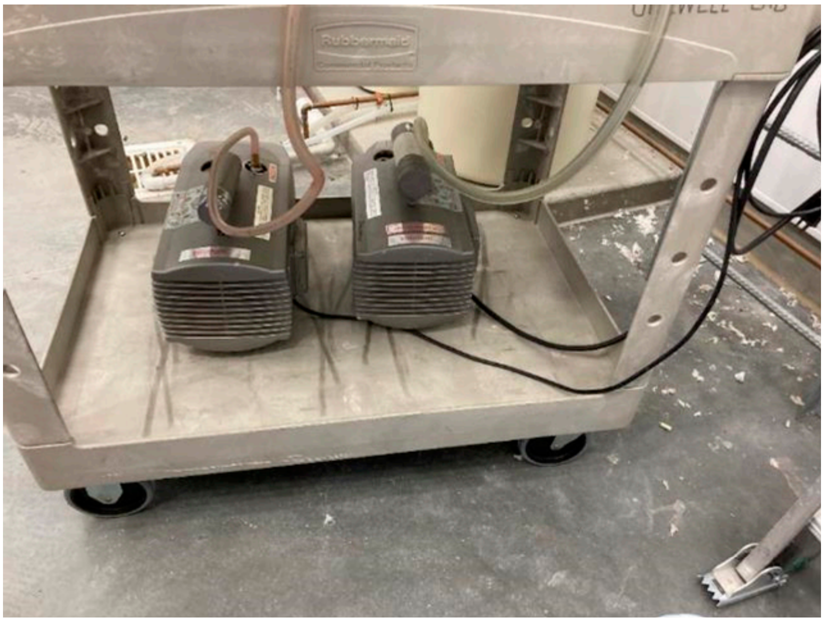

Figure S24. Two vacuum pumps (one for the inlet and one for treated air) were used to power the air sampling through the BioSamplers®.

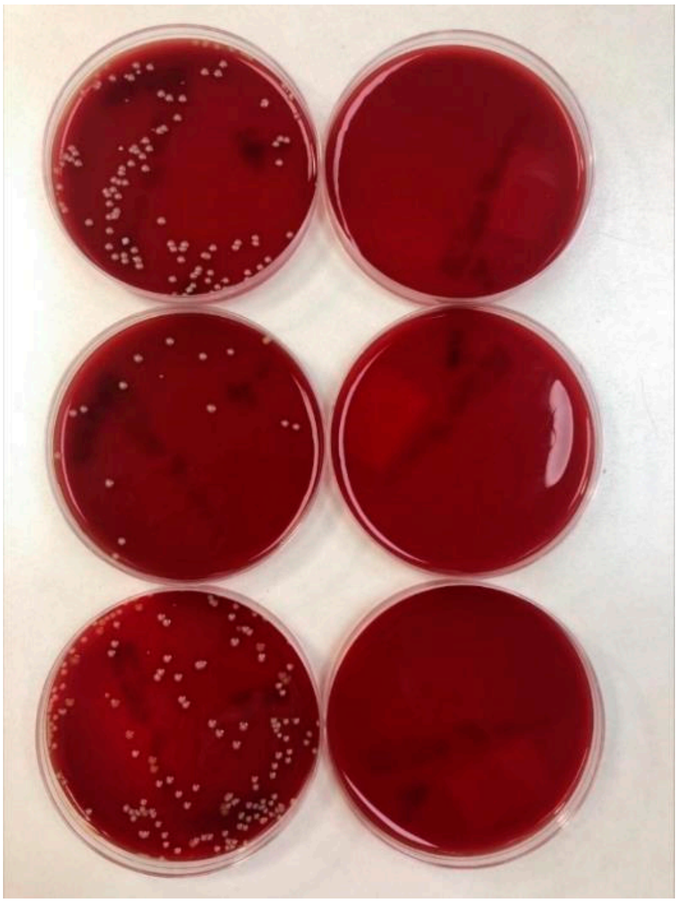

Figure S25. The results of incubation after 24 h of inoculation of the BioSampler® fluid. The three left plates showed aerobic growth (inlet), while the three right plates showed no aerobic growth (treated air). The final plate counts started after 48 h of incubation.

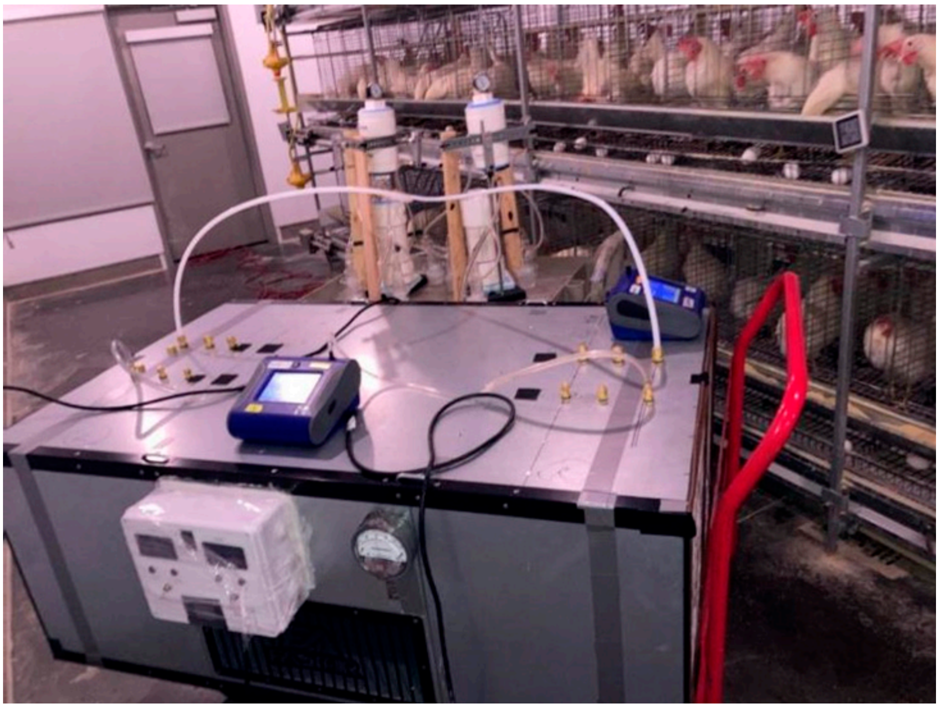

Figure S26. Details of PM measurements. Two DustTrak monitors (blue) are shown in the photo. The closer (left) one connects with both sides of the sampling ports on top of the FastAir prototype immediately after MERV-8 filters (treated air). The farther (right) one directly measures the PM concentrations in the air (inlet).

2. Supplementary Tables

Table S1. Summary of air flow rate measured by the FANS unit of the FastAir device under different conditions. These raw air flow rates were converted to standard air flow rates, and the results can be seen in Table 1 in the main text. *CFM: cubic feet per cubic meter, a customary unit commonly used in the ventilation industry in the United States.

	**Flow Rate Mode**	**Conditions (load)**	**Measured air flow rate**	
			**m^3/s**	**CFM***
**Original**	Low	No filters	0.89	1,892
		MERV 13 only	0.63	1,339
		MERV 8 & 13	0.59	1,252
	High	No filters	1.33	2,821
		MERV 13 only	1.12	2,379
		MERV 8 & 13	1.06	2,239
**Post-upgrade**	Low	UV light only	0.55	1,164
		UV light + Al mesh	0.53	1,123
		UV light + Al mesh + MERV 13	0.51	1,082
		UV light + Al mesh + MERV 8&13	0.51	1,072
	High	UV light only	1.25	2,647
		UV light + Al mesh	1.20	2,546
		UV light + Al mesh + MERV 13	1.06	2,248

		UV light + Al mesh + MERV 8&13	1.03	2,192

Supplementary Tables 2 and 3 summarize the results of the preliminary testing in a laboratory environment where the room air was very clean, and the airborne pathogen and PM concentrations were too low to be used to prove the air cleaning effectiveness and compare between the inlet and treated air.

Table S2. Testing of the FastAir prototype in a clean laboratory environment. Simultaneous sampling of Control (n = 3) and Treatment (n = 3) was conducted for airborne pathogens for 1 h. The results are expressed in CFU/mL.

Trial	Treatment Option	Inlet				Treated Air			
		No. 1	No. 2	No. 3	Mean of Inlet Air	No. 4	No. 5	No. 6	Mean of Treated Air
1	MERV-8	0	0	0	0	10	0	0	3
2	MERV-8	0	0	0	0	0	0	0	0
3	MERV-8&13	0	40	0	13	0	0	0	0
4	MERV-8&13	0	0	0	0	0	0	0	0
5	Filtration + UV	0	0	0	0	0	0	0	0

Table S3. Testing of the FastAir prototype in a clean laboratory environment. Real-time PM concentrations were measured by DustTrak monitors on the inlet and treated air for 1 h, simultaneously with the airborne bacteria sampling mentioned in Supplementary Table 1. The PM concentrations are expressed in μg/m^3.

Trial	Configuration Option	Inlet Air (μg/m^3)					Treated Air (μg/m^3)				
		PM_1	$PM_{2.5}$	PM_4	PM_{10}	TSP	PM_1	$PM_{2.5}$	PM_4	PM_{10}	TSP
1	MERV-8	1	1	1	2	3	6	6	6	6	6
2	MERV-8	5	5	5	6	8	8	8	8	9	9
3	MERV-8&13	8	8	8	8	8	8	8	8	8	8
4	MERV-8&13	1	1	1	1	2	3	3	3	3	3
5	Filtration + UV	2	2	2	2	6	4	4	4	4	5
